# Active surveillance and genetic evolution of avian influenza viruses in Egypt, 2016–2018

**DOI:** 10.1080/22221751.2019.1663712

**Published:** 2019-09-17

**Authors:** Ahmed Kandeil, Joseph T. Hicks, Sean G. Young, Ahmed N. El Taweel, Ahmed S. Kayed, Yassmin Moatasim, Omnia Kutkat, Ola Bagato, Pamela P. McKenzie, Zhipeng Cai, Rebecca Badra, Mohamed Kutkat, Justin Bahl, Richard J. Webby, Ghazi Kayali, Mohamed A. Ali

**Affiliations:** a Center of Scientific Excellence for Influenza Viruses, Water Pollution Research Department, National Research Centre, Giza, Egypt; b University of Texas Health Sciences Center, Houston, TX, USA; c Center for the Ecology of Infectious Diseases, University of Georgia, Athens, USA; d University of Arkansas for Medical Sciences, Little Rock, AR, USA; e St. Jude Children’s Research Hospital, Memphis, TN, USA; f Georgia State University, Atlanta, GA, USA; g Human Link, Hazmieh, Lebanon; h Poultry Diseases Department, National Research Centre, Giza, Egypt

**Keywords:** Avian influenza, surveillance, poultry, Egypt, genetic evolution

## Abstract

Egypt is a hotspot for avian influenza virus (AIV) due to the endemicity of H5N1 and H9N2 viruses. AIVs were isolated from 329 samples collected in 2016–2018; 48% were H9N2, 37.1% were H5N8, 7.6% were H5N1, and 7.3% were co-infections with 2 of the 3 subtypes. The 32 hemagglutinin (HA) sequences of the H5N1 viruses formed a well-defined lineage within clade 2.2.1.2. The 10 HA sequences of the H5N8 viruses belonged to a subclade within 2.3.4.4. The 11 HA of H9N2 isolates showed high sequence homology with other Egyptian G1-like H9N2 viruses. The prevalence of H5N8 viruses in ducks (2.4%) was higher than in chickens (0.94%). Genetic reassortment was detected in H9N2 viruses. Antigenic analysis showed that H9N2 viruses are homogenous, antigenic drift was detected among H5N1 viruses. AI H5N8 showed higher replication rate followed by H9N2 and H5N1, respectively. H5N8 was more common in Southern Egypt, H9N2 in the Nile Delta, and H5N1 in both areas. Ducks and chickens played a significant role in transmission of H5N1 viruses. The endemicity and co-circulation of H5N1, H5N8, and H9N2 AIV coupled with the lack of a clear control strategy continues to provide avenues for further virus evolution in Egypt.

## Introduction

Avian influenza virus (AIV) is a diverse viral pathogen maintained in wild birds and exists in high pathogenic (HPAI) and low pathogenic (LPAI) forms. Since the first detection of HPAI H5N1 subtype in 1996, the virus has evolved into 10 genetically-defined clades (0–9) and has spread throughout the world. More recently, the H5N1 viruses have undergone reassortment with other AIV and exchanged the N1 gene for other serotypes of neuraminidase to generate different subtypes of H5NX viruses [1,2].

H5N1 viruses of clade 2.2.1 have been widely circulating in Egypt since 2006, resulting in massive economic losses for the Egyptian poultry industry and causing public health concerns [3]. Clades 2.2.1.1, 2.2.1.1a, and 2.2.1.2 of Egyptian H5N1 viruses evolved from the parent clade 2.2.1 [4]. Clades 2.2.1 and 2.2.1.1 viruses co-circulated from 2009 through 2014. It has been hypothesized that clade 2.2.1.1 viruses emerged as vaccine escape mutants due to vaccine application [5]. Further evolution of these viruses led to a new phylogenetic cluster, clade 2.2.1.2 [4]. In winter 2016, the H5N8 subtype of clade 2.3.4.4 (group B) was detected in migratory wild birds in two Mediterranean regions of Egypt [6,7]. Since then, several H5N8 outbreaks have been detected in domestic poultry in several governorates in Egypt [8]. Despite this wide dissemination of H5N8 viruses, there have been no reports of associated human infections.

Between 2010 and 2015, H9N2 viruses of the G1-lineage were isolated from chickens, ducks, turkeys, and quails in Egypt [9,10]. Infected poultry showed no clinical illness or at worst mild respiratory signs. Surveillance showed frequent H5N1/H9N2 co-infection but reassortants were not detected, unlike reports from Asia [11–13]. Kandeil et al. detected novel reassortant H9N2 viruses from pigeons in Egypt that had five genes from Eurasian AIVs circulating in wild birds with HA, NA, and M genes from the endemic H9N2 viruses [14].

The dynamic ecology and epidemiology of AIV in poultry in Egypt necessitate and highlight the value of long term, longitudinal surveillance programmes. Our active surveillance for AIV in Egypt has been continuously running since 2009 with periodic reports being published [11,15–17]. Despite sustained monitoring and control efforts, HPAI and LPAI viruses continue to circulate increasing the emergence risk of novel variants with pandemic potential. Here, we integrate multiple data sources to (1) provide an update on the situation of circulating AIVs in Egypt from January 2016 to December 2018, (2) develop an ecological niche model of all co-circulating influenza subtypes to understand how environmental factors can impact the distribution of variants, and (3) investigate the impact of viral transmission in a multi-host system and the effect on HPAI virus persistence and diversity of variants within Egypt.

## Methods

### Collection of samples

Between January 2016 and December 2018, 6137 cloacal and 5073 oropharyngeal swabs were collected from birds in 39 commercial poultry farms, 22 backyard flocks, 2 abattoirs, and 22 live-bird markets in Egypt as part of an existing virological surveillance programme. Sampling was performed in 4 Nile Delta governorates [Dakahliya (3 backyard flocks, 4 farms, and 3 markets), Monofiya (3 farms), Kalyobiya (1 backyard flock, 5 farms, and 1 market), and Sharqeia (3 backyard flocks, 4 farms, and 3 markets)]; Fayoum in middle Egypt(1 backyard flock, 5 farms, 1 abattoir, and 1 market); and 4 Southern Egypt governorates [Assiut (4 backyard flocks, 5 farms, and 5 markets), Bani Souwaif (2 farms), Menia (5 backyard flocks, 3 farms, 1 abattoir, and 5 markets), and Sohag (5 backyard flocks, 8 farms, and 5 markets)]. Samples were collected from healthy, sick, and dead birds (chickens [*n* = 8793], ducks [*n* = 1348], geese [*n* = 80], pigeons [*n* = 716], turkeys [*n* = 165], and quails [*n* = 108]). All samples were collected and stored as previously described [17]. Ethical approval was obtained from the Ethics Committee at the National Research Centre, Egypt.

### Virus isolation and subtyping

All collected samples were individually inoculated in the allantoic cavities of 10-day-old specificpathogen-free embryonated hens’ eggs and incubated for up to two days and checked daily for embryo death. Eggs with dead embryos were chilled under 4°C. Then, 100 µl of each allantoic fluid were tested for hemagglutinating activity using 0.5% chicken red blood cells [18]. The positive samples were subjected to viral RNA extraction using QIAamp viral mini kit (Qiagen, Germany) according to the manufacturer’s protocol, then typed as positive or negative for influenza virus by using a real time RT-PCR assay for the M-gene [19]. The positive M-gene samples were further HA and NA subtyped as previously described [11,20]. The proportions of positive samples were calculated across various characteristics, including sample type, governorate, host species, poultry production type, and bird health status. Significant differences between proportions were tested by the Pearson’s chi-squared test or Fisher’s exact test using SPSS v23 (IBM, Armonk, NY). A *p*-value ≤ 0.05 was considered statistically significant.

### Amplification of viral genome, sequence analysis, and phylogenetic tree construction

First-strand cDNA was synthesized from extracted RNA using Superscript III Reverse transcriptase (Invitrogen, Carlsbad, CA) and Uni-12 primer (5′AGCRAAAGCAGG3′) as per manufacturer’s protocol. Using Phusion Master Mix kit (Thermo Scientific, Wilmington, USA), the desired viral genes were amplified using universal primers [21]. Briefly, using gene-specific primers, 2 µl of each reaction were subjected to PCR by an initial denaturation step (98°C for 30 s), followed by 40 cycles each consisting of 98°C for 10 s, 57°C for 30 s, 72°C for 3 min, and a final elongation at 72°C for 10 min. Amplicons of the appropriate sizes were gel purified using QIAGEN gel purification kit (Qiagen). The purified PCR products were sequenced at Macrogen sequencing facility (Macrogen, South Korea). Sequences were assembled using SeqManDNA Lasergene 7 software (DNASTAR, Madison, WI). Sequenced genes and their accession numbers are shown in Table S2.

Publicly available HA gene sequences from HPAI H5 viruses from all avian hosts collected between 1 January 2005 and 1 September 2018 were downloaded from the Global Initiative on Sharing All Influenza Data (GISAID) (http://platform.gisaid.org/epi3/) on 12 September 2018. HA genes were aligned with the newly sequenced Egyptian virus genes using Muscle v3.8 [22]. Duplicate sequences were removed. Due to the large number of sequences, a computationally light neighbour joining tree method (Paup^∗^ v4.0) was used to identify genetically distant viral lineages that did not share direct ancestry with the newly sequenced Egyptian samples [23]. These distantly related sequences were removed. For the HA H5 sequences, BEAST v1.8 was used to estimate a Bayesian maximum clade credibility phylogeny with a general time reversible (GTR) nucleotide substitution model, a gamma distribution of substitution rates, a proportion of invariant nucleotide sites, a Gaussian Markov random field (GMRF) Bayesian skyride coalescent model, and a lognormal uncorrelated relaxed molecular clock [24]. The three independent Markov Chain Monte Carlo simulations were allowed to run for a length of 100 million states each, sampling every 10,000 states. At least a 10% burn-in was removed from each run to ensure an adequate effective sampling size (ESS) > 200 for all estimated parameters. Tree nodes with a posterior probability > 0.95 are considered well-supported. Phylogenetic trees of H9, internal segments, N1, N2, and N8 sequences were performed with BEAST v1.8 in the manner described above. BioEdit programme version 7.0 was used for genomic signature analysis.

### Antigenic analysis

A panel of anti-H5 monoclonal antibodies (mAbs) including six (VN04-2, VN04-8, VN04-9, VN04-10, VN04-13 and VN04-16) generated to A/Viet Nam/1203/04 (H5N1) and two (BHG05-1 and BHG05-2) generated to A/bar-headed goose/QH/1A/05 (H5N1) were used to antigenically characterize the different virus isolates using hemagglutination inhibition (HI) assay.

Antigenic analyses of the H9N2 viruses were performed by HI assay using chicken antisera generated by vaccinating chickens with different H9N2 antigens as previously described [25]. These antisera were used for antigenic mapping of Egyptian H9N2 viruses based on the differences in HI titres using the integrative matrix completion multi-dimensional scaling (MC-MDS) method as previously described [26,27]. Matrix completion was used to remove the data noise in the HI experiment. Multi-dimensional scaling projected the antigens onto a two-dimensional grid.

### Propagation rates of AIVs

The replication rates of plaque-purified egg-cultured A/chicken/Egypt/Q1995D/2010(H5N1, clade 2.2.1.1), A/chicken/Egypt/F12505C/2016(H5N1, clade 2.2.1.2), A/duck/Egypt/F13663A/2017(H5N8), and A/chicken/Egypt/F13454A/2016(H9N2) viruses were compared. A dilution of 10^4^ egg infective dose (EID_50_) of each virus was prepared in phosphate buffered saline (PBS) and a volume of 0.1 mL of each virus suspension was individually inoculated into three specific-pathogen-free embryonated chicken eggs. Inoculated eggs were incubated at 37°C for 24 h post-infection then chilled at 4°C for 4 h. Allantoic fluids of infected eggs were collected and used for inoculation of two subsequent passages of embryonated chicken eggs without dilution. The growth dynamics of the viruses at all passages were evaluated by comparing titres obtained using a hemagglutination assay with 0.5% chicken RBCs and by comparing the number of RNA copies of each virus at each passage. Viral RNA was extracted from each harvest using the viral RNA Mini kit (Qiagen) and subjected to qRT-PCR targeting the M-gene.

### Ecological niche modelling

To compare environmental conditions associated with the emergence of each virus subtype, we performed ecological niche modelling (ENM) using the maximum entropy algorithm, a conservative presence-only algorithm shown to be robust to overfitting [28–30]. ENM predicts the potential geographic distribution of organisms (in this case influenza viruses) based on previously observed occurrences in relation to relevant environmental variables representing the ecological niche. Based on previous modelling efforts, we selected environmental datasets with demonstrated impact on viral and host persistence, transmission, and diffusion including distance to fresh water, human population (a proxy for poultry), temperature, precipitation, and humidity [31,32].

Fresh water and population data were obtained from the Defense Mapping Agency’s Digital Chart of the World and NASA SEDAC’s Gridded Population of the World v4 [33,34]. The remainder of the environmental variables were obtained from NASA’s GLDAS Noah Land Surface Model dataset, collected continuously via satellites from 2005 to 2016 and temporally averaged from monthly to bi-annual measurements at a spatial resolution of 0.25° [35,36]. In contrast with previous studies, we focused our modelling efforts on the first year of emergence for each viral subtype. Niche models were built using outbreak data from the first full year of emergence, with environmental variables from corresponding years; data from 2005 to 2006 was used for H5N1, 2010–2011 for H9N2, and 2015–2016 for H5N8. Newly emerging epidemic subtypes tend to diffuse rapidly in naïve populations, spreading evenly into areas of marginal suitability before spatially contracting over time to a more suitable niche, i.e. transitioning from epidemic to endemic. By limiting our analyses to the first year of emergence we intentionally overestimate the suitable area for each subtype to emphasize their similarities and differences during emergence. Niche estimates were mapped using ArcGIS 10.6 (Esri, Redlands, CA, USA) and distributions were compared visually and statistically using Warren’s I similarity statistic based on Hellinger Distances, calculated with the phyloclim (v 0.9-5) package in R v3.5.0, (R Core Team, Vienna, Austria) [37,38]. To evaluate if observed differences in subtype distribution could be attributed to variable environmental conditions between years of emergence, niche models for H9N2 and H5N8 were then projected onto environmental data from 2006 (corresponding to the H5N1 model) and new niche estimates were created and compared to previous estimates.

### Host discrete trait diffusion model

To further investigate the role of avian host in the evolution of H5N1 subtype HA in Egypt, a discrete trait diffusion model was conducted in BEAST v1.8 using a supported H5N1 Egyptian clade as determined by the full H5 HA phylogenetic analysis. The goal of this analysis was to reveal patterns of viral transmission among hosts within Egypt and to determine which hosts have been associated with viral movement into and out of the country. Discrete trait diffusion models assume viral traits, such as host, “evolve” along the phylogenetic history, allowing the use of substitution model-based methods to infer the host history among ancestral viruses. Sequences were categorized by reported host of viral isolation (chicken, duck, goose, and turkey). Rare hosts were combined into a single “other host” category. In addition, sequences isolated from hosts sampled outside Egypt were combined into a single category. To mitigate the influence of the large amount of chicken surveillance in Egypt, the dataset was randomly subsampled so that no host category had more than five sequences per year. A BEAST analysis then was performed using the same phylogenetic parameters as the full H5 HA analysis, but with the further specification of an asymmetric discrete trait diffusion model [39]. Bayesian stochastic search variable selection (BSSVS) was employed to estimate statistical support for transition rates among the host categories [2]. A transition rate was considered supported when both the Bayes Factor (BF) > 3 and the posterior probability (pp) > 0.5. BF support can be further qualified with the following categories: substantial support (3.0 < BF ≤ 10.0), strong support (10.0 < BF ≤ 30.0), very strong support (30.0 < BF ≤ 100.0), and decisive support (BF > 100.0). BF and pp were calculated in SPREAD3 [40]. To assess the influence of sampling bias, a sensitivity analysis was performed in which the host assignments continually changed throughout the discrete trait diffusion model estimation. In this way, the host proportions within the sample remain constant providing an assessment of the impact of oversampling on observed host transition rates.

## Results

### Surveillance of AIV in poultry

Of 11,210 poultry samples collected between January 2016 and December 2018, 329 (2.93%) were positive for AIV by virus culture (Table 1). The isolation rate in embryonated chicken eggs was two times higher in oropharyngeal swabs than cloacal swabs (*p* < 0.001). Detection rates differed significantly by governorate, host species, and poultry production sector (*p* < 0.001). No significant difference by bird health status was observed (*p* > 0.05). Of the governorates in the Nile Delta region, Dakahliya showed the highest prevalence (2.2%). In Southern Egypt, the highest prevalence was found in Menia governorate (4.9%). Among hosts, chickens showed the highest prevalence (3.3%), followed by ducks (3%), geese (1.2%), quails (0.9%), and pigeons (0.6%). No virus was detected in samples from turkeys. The prevalence was highest in live bird markets (3.8%) compared to backyard flocks (3.5%) and commercial farms (2.3%). No virus was detected in samples from abattoirs. Although not statistically significant, more positive samples were detected in sick birds (5.3%) than in healthy (2.9%) or dead birds (2.6%).

**Table 1.** Epizootic data of avian influenza in Egypt.

Variable	No. Collected Samples (%^a^)	No. of Influenza A-positive Samples (%^b^)	Subtype (%^c^)					
			H5N1	H9N2	H5N8	H5N1/H9N2	H5N8/H5N1	H5N8/H9N2
*Sample Type*	* *	* *	* *	* *	* *	* *	* *	* *
Cloacal	6137 (54.8)	121 (2.0)^d^	12 (9.9)	38 (31.4)	62 (51.2)	3 (2.5)	3 (2.5)	3 (2.5)
Oropharyngeal	5073 (45.2)	208 (4.1)	13 (6.2)	120 (57.7)	60 (28.9)	6 (2.9)	7 (3.3)	2 (1.0)
*Governorate*	* *	* *	* *	* *	* *	* *	* *	* *
Dakahliya	1888 (16.8)	41 (2.2)^d^	1 (2.4)	37 (90.3)	1 (2.4)	0 (0)	0 (0)	2 (4.8)
Kalyobiya/Monofiya	1135 (10.1)	21 (1.8)	3 (14.3)	3 (14.3)	14 (66.7)	1 (4.7)	0 (0)	0 (0)
Sharqeia	2049 (18.3)	31 (1.5)	5 (16.2)	26 (83.8)	0 (0)	0 (0)	0 (0)	0 (0)
Fayoum/Banisouwaif	1797 (16)	59 (3.2)	10 (16.9)	18 (30.5)	24 (40.6)	4 (6.8)	1 (1.7)	2 (3.4)
Sohag	880 (7.9)	9 (1.0)	0 (0)	0 (0)	9 (100)	0 (0)	0 (0)	0 (0)
Assiut	1546 (13.8)	73 (4.7)	0 (0)	50 (68.5)	20 (27.4)	1 (1.3)	1 (1.3)	1 (1.3)
Menia	1915 (17.1)	95 (4.9)	6 (6.3)	24 (25.2)	54 (56.8)	3 (3.1)	8 (8.4)	0 (0)
*Species*	* *	* *	* *	* *	* *	* *	* *	* *
Chickens	8793 (78.43)	288 (3.3)^e^	25 (8.7)	155 (53.8)	86 (29.9)	9 (3.1)	8 (2.8)	5 (1.7)
Ducks	1348 (12.02)	35 (3.0)	0 (0)	0 (0)	33 (94.3)	0 (0)	2 (5.7)	0 (0)
Geese	80 (0.7)	1 (1.2)	0 (0)	0 (0)	1 (100)	0 (0)	0 (0)	0 (0)
Pigeons	716 (6.4)	4 (0.6)	0 (0)	2 (50)	2 (50.0)	0 (0)	0 (0)	0 (0)
Turkeys	165 (1.48)	0 (0)	0 (0)	0 (0)	0 (0)	0 (0)	0 (0)	0 (0)
Quails	108 (0.96)	1 (0.9)	0 (0)	1 (100)	0 (0)	0 (0)	0 (0)	0 (0)
*Location*	* *	* *	* *	* *	* *	* *	* *	* *
Abattoir	80 (0.7)	0 (0)^e^	0 (0)	0 (0)	0 (0)	0 (0)	0 (0)	0 (0)
Commercial farm	6202 (55.3)	145 (2.3)	18 (12.4)	70 (48.2)	48 (33.1)	5 (3.4)	2 (1.4)	2 (1.4)
Backyard flock	2432 (21.7)	87 (3.5)	6 (6.9)	19 (21.8)	56 (64.4)	1 (1.1)	5 (5.7)	0 (0)
Live bird market	2496 (22.2)	97 (3.8)	1 (1)	69 (71.1)	18 (18.5)	3 (3.1)	3 (3.1)	3 (3.1)
*Bird Health Status*	* *	* *	* *	* *	* *	* *	* *	* *
Healthy	8807 (78.6)	260 (2.9)	13 (5)	136 (52.3)	92 (35.4)	4 (1.5)	10 (3.8)	5 (1.9)
Sick	168 (1.4)	9 (5.3)	5 (55.5)	4 (44.4)	0 (0)	0 (0)	0 (0)	0 (0)
Dead	2235 (20.0)	60 (2.6)	7 (11.6)	18 (30)	30 (50.0)	5 (8.4)	0 (0)	0 (0)

^a^% of total samples collected;

^b^% of samples in category;

^c^% subtype positive within influenza A positive samples in category;

^d^Statistically significant by Chi-square test;

^e^Statistically significant by Fisher exact test.

The distribution of subtypes by species, bird health status, poultry production sector, and governorate is shown in Table 1. Of the 329 isolated viruses, 48% were H9N2, 37.1% were H5N8, 7.6% were H5N1, and 7.3% were co-infections with 2 of the 3 virus subtypes. H5N1 was only detected in chickens, mostly in the Nile Delta and Fayoum. The prevalence of H5N8 viruses in ducks (2.4%) was higher than in chickens (0.94%). While H5N8 viruses were detected in backyard flocks, commercial farms, and live bird markets in all regions, they were more commonly found in Southern- and Middle Egypt. H9N2 was most common in chickens in all regions of Egypt.

**Figure 1.**
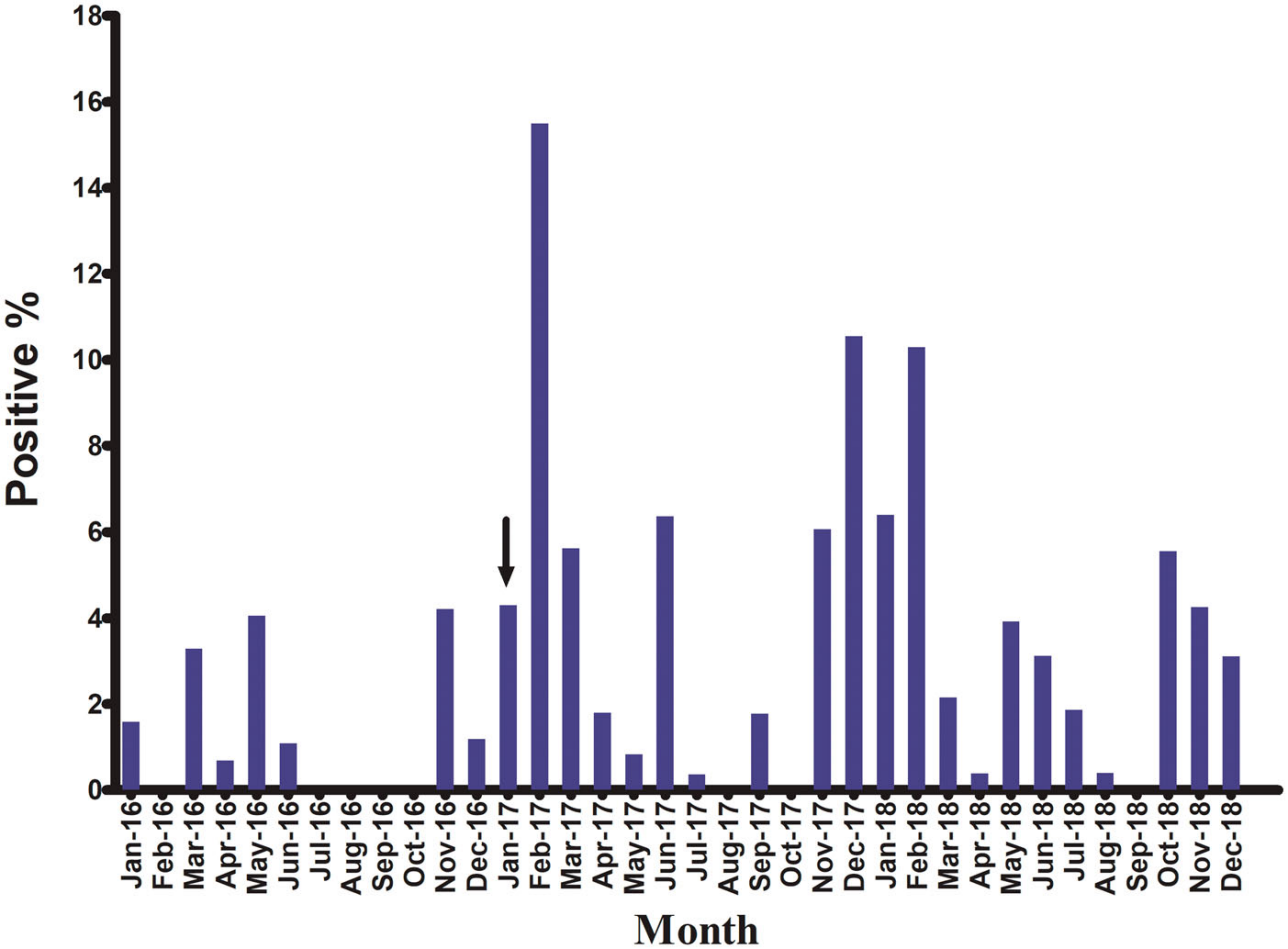
Monthly isolation rates of influenza A viruses detected in poultry, 2016–2018. Arrow indicates when H5N8 was first detected in Egyptian poultry.

**Figure 2.**
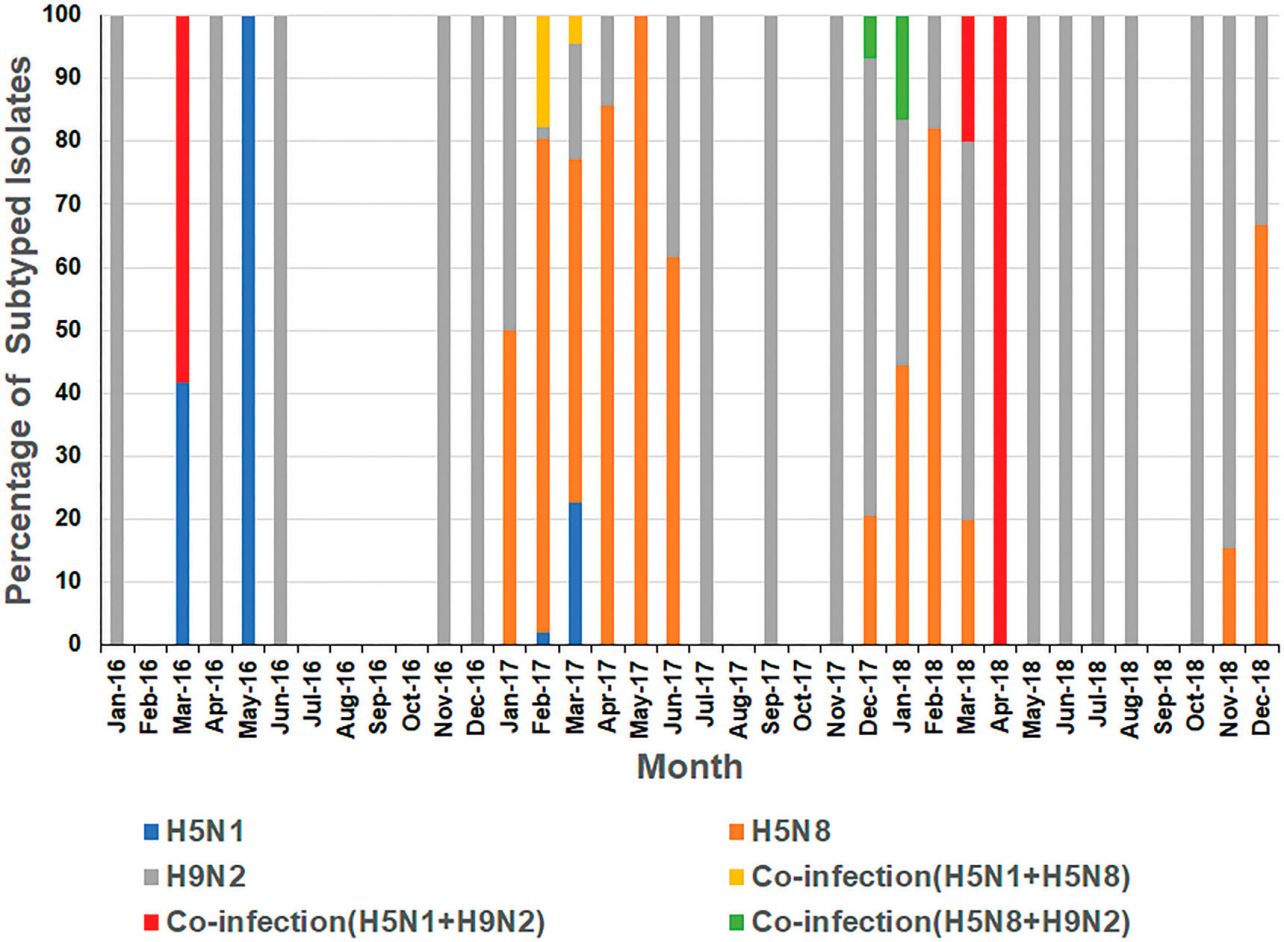
Monthly distribution of avian influenza A viruses in Egyptian poultry, 2016–2018.

Most positive samples were detected in colder months of the year (Figure 1). During 2016, both H5N1 and H9N2 viruses were detected with H5N1 positivity peaking in May 2016, and H9N2 peaking in November 2016 (Figures 1 and 2). No viruses were detected in samples collected from July to October 2016. H5N8 viruses were not detected in poultry until January 2017 when they were detected in apparently healthy chickens and ducks in backyard flocks in Fayoum. During 2017, H5N1, H5N8, and H9N2 were detected with positivity peaking in February 2017 due to increased H5N8 infections (Figures 1 and 2). Co-infection (H5N1/H5N8) was recorded in February and March 2017 in Southern Egypt (Assiut and Menia) and in middle Egypt (Fayoum), while H5N8/H9N2 co-infection was recorded only in December 2017 in Dakahliya governorate. In 2018, H5N8, H9N2, and co-infections (H5N8/H5N1 and H5N1/H9N2) were detected. No H5N1 virus was detected in samples collected after April 2018 (Figure 2).

### Genetic analysis of influenza A viruses

#### H5N1

**Figure 3.**
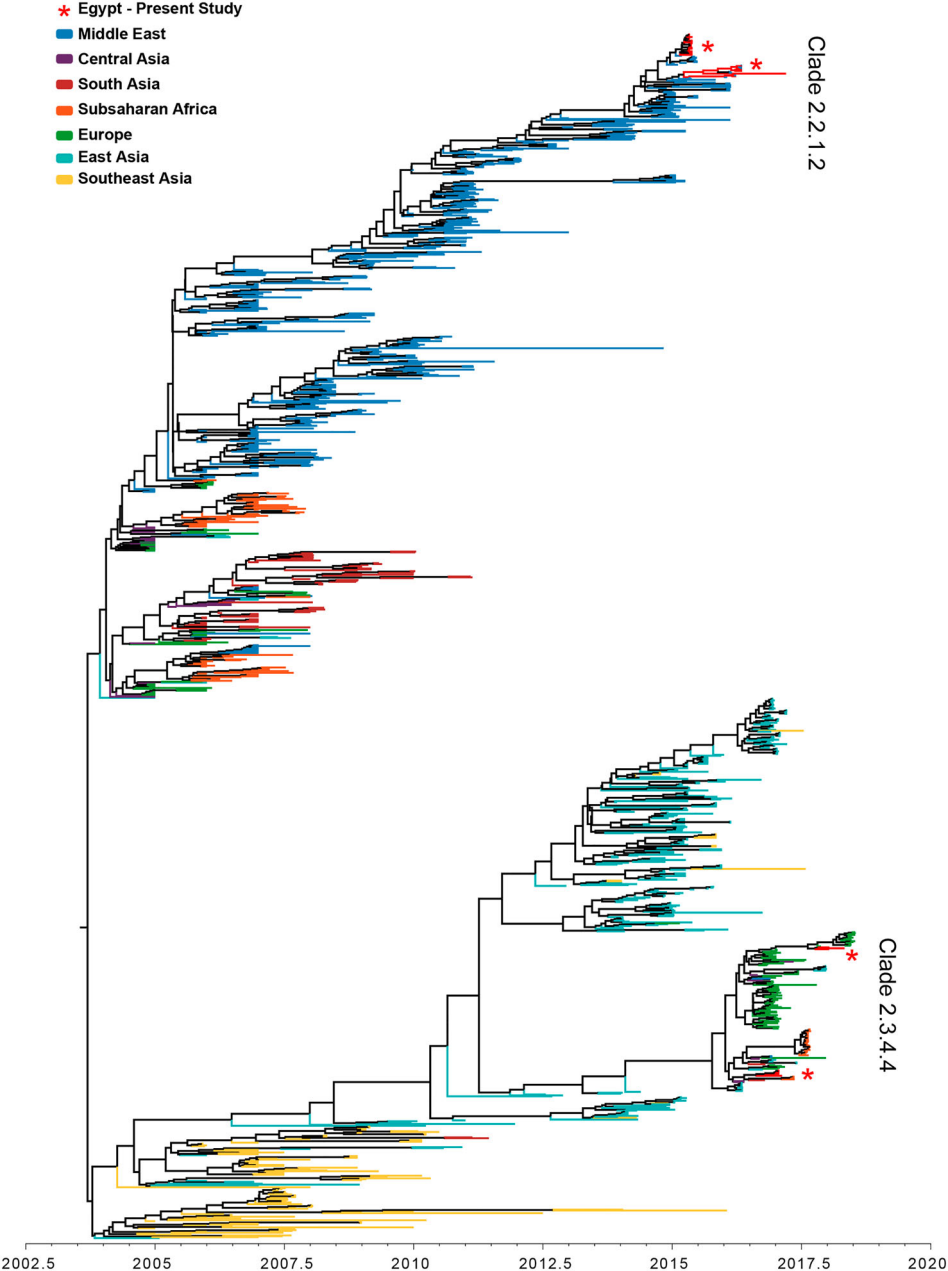
Phylogenetic tree of the nucleotide sequences of H5 genes of characterized from Egypt, Europe, Central Asia, the Middle East, Sub-Saharan Africa, and South Asia collected between January 1, 2005 and September 2018.

A total of 1,351 HA sequences from the Middle East, Europe, Sub-Saharan Africa, and Asia were included in the Bayesian phylogenetic analysis, including 32 newly sequenced H5N1 HA isolates from 2015 to 2018. The analyzed sequences share a most recent common ancestor 14.9 years ago (95% HPD 14.6–15.1 years ago), and they are visually divided into two main lineages (Figure 3). The majority of Egyptian sequences reside in a well-defined Middle Eastern lineage of clade 2.2.1 that diverged from other H5 sequences 13.5 years ago (95% HPD 13.2–13.9 years ago). This Middle Eastern lineage had a strong ladder-like morphology indicating immune selective pressure. The 32 newly sequenced Egyptian H5N1 HA sequences exist within the Middle Eastern lineage, 24 of which exist in a supported monophyletic clade (posterior = 1.0), suggesting they originate from the same outbreak. A subsampled Bayesian phylogenetic analysis of the Middle Eastern lineage revealed a median nucleotide substitution rate of 0.0044 substitutions per site per year (95% HPD 0.0039–0.0049) (Table S1). Phylogenetically, the characterized H5N1 viruses belonged to the clade 2.2.1.2 clustering with gene sequences of viruses isolated in 2014 and 2015 from Egyptian poultry (Figures 3). All Egyptian H5N1 viruses possess the polybasic amino acids at the HA cleavage site PQGEKRRKKR/G. All viruses possessed six N-linked glycosylation sites at amino acid positions 10, 23, 165, 286, 483, and 542 (H5 numbering), except for A/chicken/Egypt/B13826D/2017(H5N1) that lost a glycosylation site at position 23 (Table S3(B)). H5N1 viruses isolated in the period from 2015 to 2017 had glutamine (Q) and glycine (G) at residues 222 and 224 (H5 numbering) respectively which are associated with preferential binding to avian like receptors. A/chicken/Egypt/B13825A/2017(H5N1) and A/chicken/Egypt/B13826D/2017(H5N1) had E127D and R140K mutations in antigenic site A in the HA glycoprotein. Reassortment of influenza A (H5N1) in Egypt was not detected (Figure S2).

#### H5N8

The 10 H5N8 HA sequences generated in this study are found in a well-supported clade containing viruses of heterogeneous geographic origin. This group includes other Middle East isolates, as well as isolates sampled in Sub-Saharan Africa, South Asia, Central Asia, East Asia, and Europe. A cluster (posterior = 1.0) of seven newly sequenced and six previously published Egyptian sequences shares a recent common ancestor with an H5 sequence from India and 4 recent H5 sequences from the Democratic Republic of Congo, diverging between 2.0 and 2.3 years ago (95% HPD) (Figures 3, S3). The remaining three newly sequenced Egyptian H5N8 HA sequences in 2018 form a monophyletic group (posterior = 1.0) within a lineage of sequences of European origin. The lineage containing all currently available Egyptian H5N8 HA sequences had a substitution rate of 0.0036 substitutions per site per year (95% HPD 0.0026–0.0051) (Table S1). All Egyptian H5N8 viruses possess the polybasic amino acids at the HA cleavage site PLREKRRKR/G.

HA genes of H5N8 viruses had six potential glycosylation sites at positions 10, 23, 165, 286, 483 and 542 (Table S3). Those had glutamine (Q) and glycine (G) at residues 222 and 224 (H5 numbering) respectively, indicating preferential binding to avian-like receptors. Mutations A140T and E156A were detected in antigenic sites A and B of A/pigeon/Egypt/A15052/2018(H5N8) and A/chicken/Egypt/F13660A/2017(H5N8), respectively. The remaining viruses had the same antigenic profile of group B of clade 2.3.4.4 viruses as previously described [6]. All other genes of three H5N8 viruses isolated in 2017 from three different governorates (Kalyobiya, Fayoum, and Menia) were identical (Figure S2).

#### H9N2

The 11 HAs of the H9N2 viruses isolated showed high sequence homology with other Egyptian H9N2 strains belonging to group B of G1-like H9N2 viruses (Figure S1). The Egyptian group B sequences share a common ancestor that existed about 8.6 years ago (95% HPD 7.6–9.7). Included H9N2 HA sequences shared a most recent common ancestor between 52–54 years ago with a median substitution rate of 0.0065 substitutions per site per year (95% HPD 0.0057–0.0074) (Table S1). The PB2, PB1, PA, and NS genes were similar to previously identified viruses in Egypt as of 2014. The other three genes were related to H9N2 viruses circulating in Egypt since 2010 (Figure S2). HAs of H9N2 viruses had ^335^RSSR^∗^GLF^341^ (H9 numbering) cleavage motif sequence, which is the signature of low pathogenicity. No significant mutations were detected in the H9 genes (Table S3).

### Antigenic Characterization of H5N1 and H9N2 Viruses

**Figure 4.**
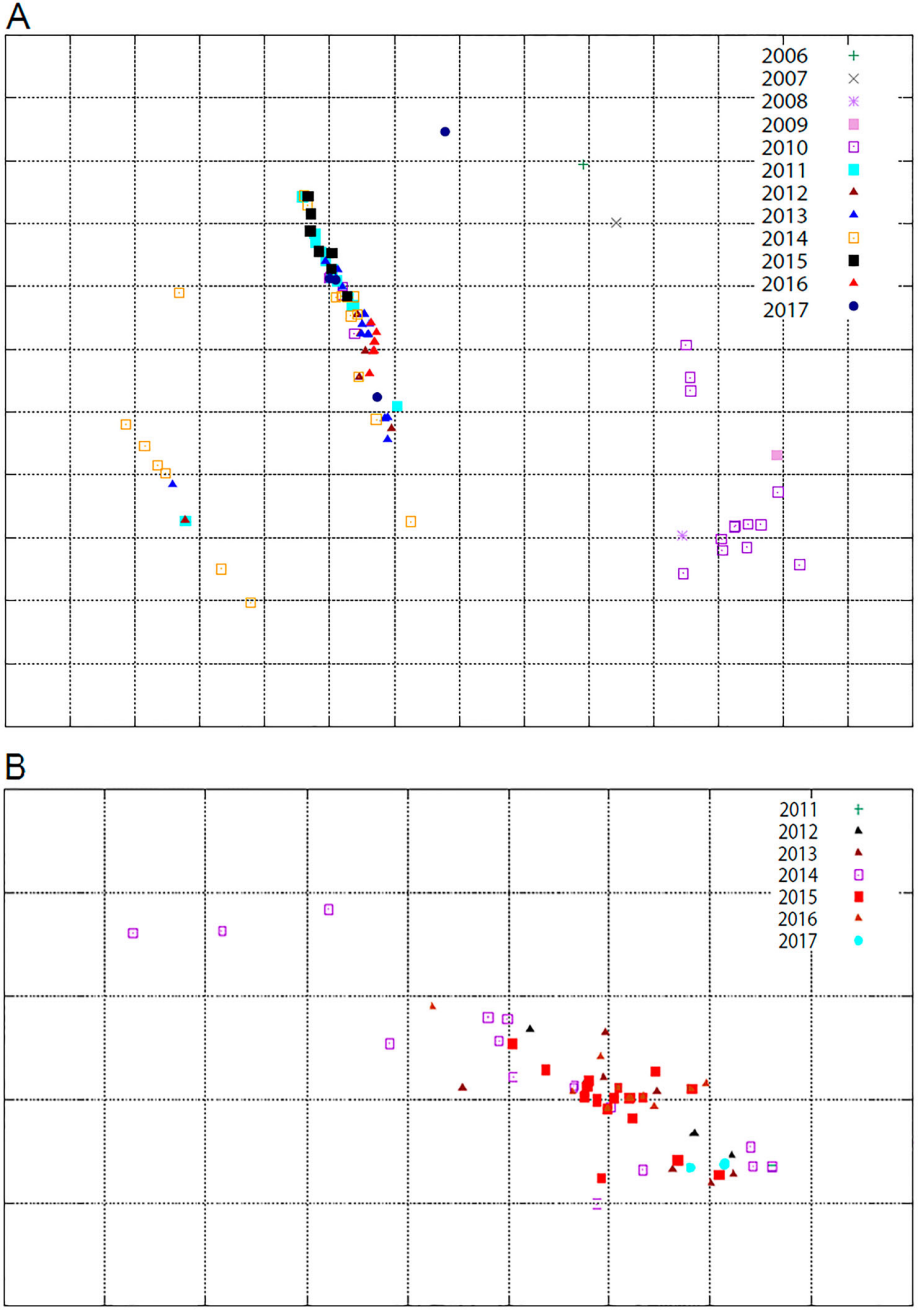
Antigenic cartography representation of the Egyptian HPAI A(H5N1) (A) and LPAI A (H9N2) isolates (B) using a panel of monoclonal and polyclonal antibodies, respectively. The maps were generated using Antigen-Map http://sysbio.cvm.msstate.edu/AntigenMap). AIV isolates of each year are distinguished by different symbols and colours.

Results of the antigenic characterization of H5N1 viruses were used to update previously published antigenic cartographies [16,41]. The HI data indicated that the H5N1 viruses have drifted over time (Figure 4(A)). A/chicken/Egypt/B13826D/2017(H5N1) had a distinct antigenic form compared to other clade 2.2.1.2 H5N1 viruses as a result of the loss of a glycosylation site at position 23 (NVTV mutated to SVTV) and the occurrence of mutations E127D, R140K, and T202A (H5 numbering) in the HA glycoprotein. Several H5N1 strains from 2013 and 2014 had a distinguishable antigenic profile due to the R140K mutation in the HA.

The antigenic characterization results of H9N2 isolates were used to update a previously published antigenic cartography [10]. The Egyptian H9N2 viruses isolated in 2016 and 2017 reacted similarly to the polyclonal antibodies as did previously characterized viruses. The cartography of the Egyptian H9N2 viruses showed that, except for drifted H9N2 viruses initially isolated from quails in 2014 [10], all viruses fell into one cluster (Figure 4(B)).

### Propagation rates

**Figure 5.**
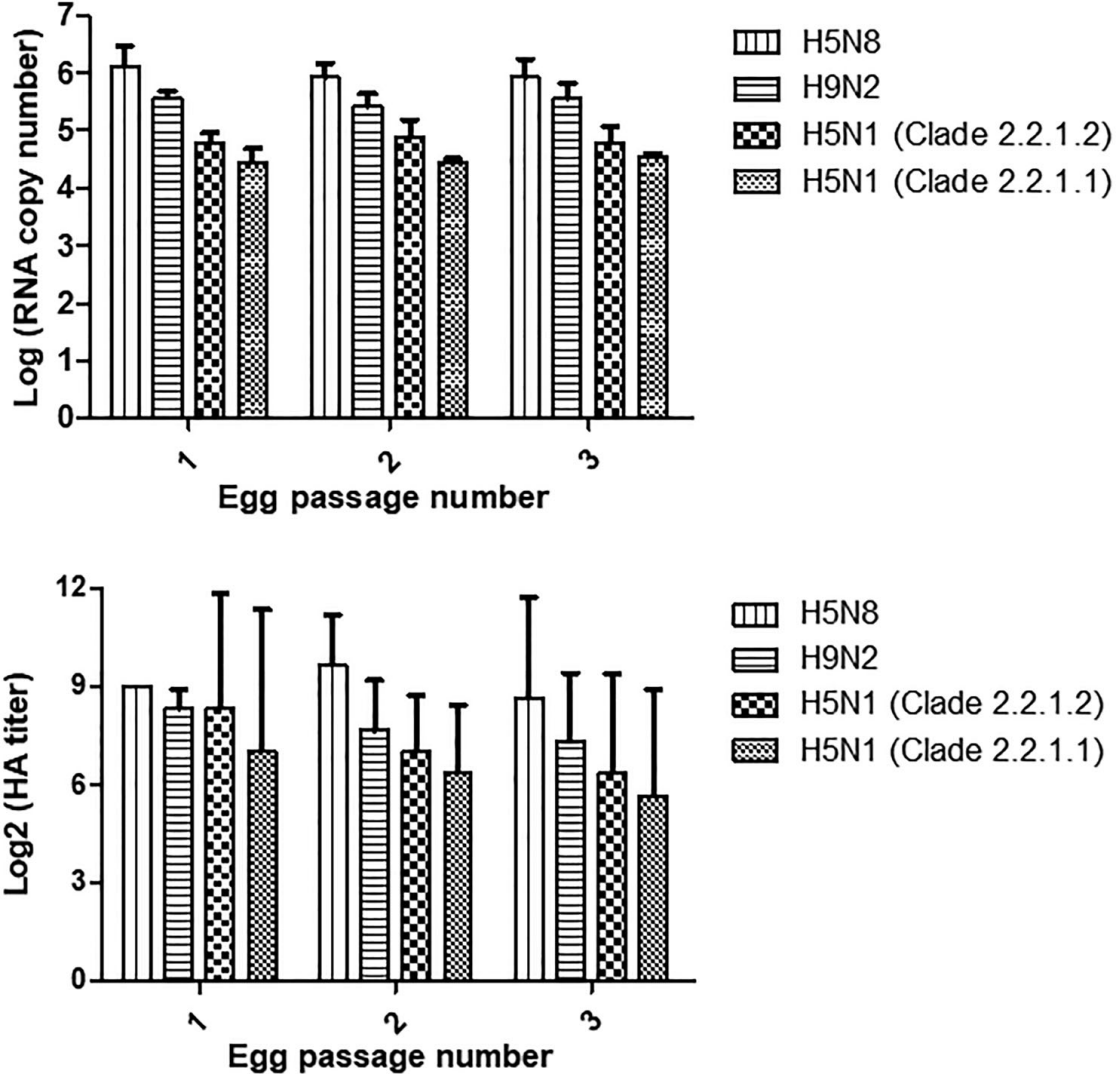
Replication rates of H5N8, H9N2, and H5N1 viruses from Egypt. Rates were assessed by comparing RNA copy numbers (top) and hemagglutination titres (bottom).

We hypothesized that viral replication rates may contribute to viral distribution in the Egyptian environment. The results in Figure 5 showed that H5N8 grew more efficiently than the other viruses with the highest HA titre and RNA copy numbers over the three successive passages. H9N2 grew more efficiently than both H5N1 viruses.

### Ecological niche modelling

**Figure 6.**
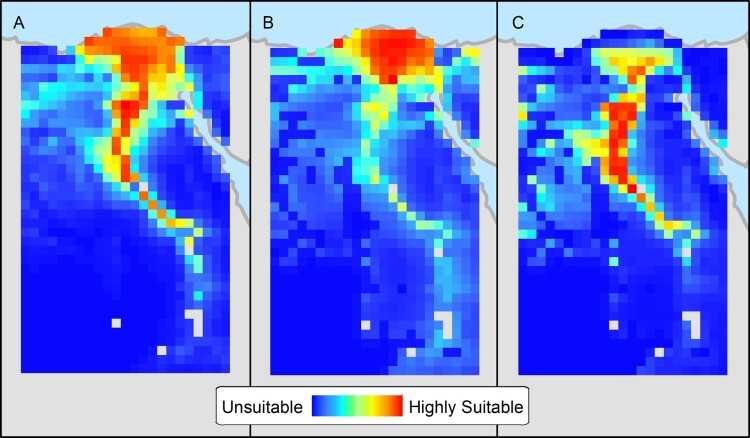
Niche estimates for the emergence of H5N1 in 2005–2006 (A), H9N2 in 2010–2011 (B), and H5N8 in 2015–2016 (C) in northern Egypt based on ecological niche models.

AUC values for the three niche models were high, indicating good predictive ability: 0.91 for H5N1, 0.94 for H9N2, and 0.95 for H5N8. Figure 6 shows the mapped niche estimates for the first year of emergence of each subtype, H5N1 (A), H9N2 (B), and H5N8 (C). Note the overall amount and location of land area that is predicted to be suitable for each viral subtype. For example, a larger area is suitable for H5N1 than the others including the Nile delta, lower Egypt, and middle Egypt. In contrast, the delta is the only area considered highly suitable for H9N2 based on 2011 data, but lower Egypt south of the delta seems to be the ideal for H5N8. Response curves for individual variables (not shown) appear to be similar between models, and Warren’s I showed substantial overlap between niche estimates (I = 0.951 for H5N1/H9N2 and I = 0.888 for H5N1/H5N8). The niche estimate derived from projecting the H9N2 model onto environmental data from 2006 was nearly identical (I = 0.997) to the niche estimate created using data from 2011. The niche estimate for H5N8 projected to 2006 was also similar (I = 0.981) to the estimate from 2016.

The similarity of ENM response curves between models and the moderately high I statistics confirm that the virus subtypes fill similar (although not identical) ecological niches. The projection of niche models for H9N2 and H5N8 onto environmental data from 2006 created niche estimates that were extremely similar to those created using 2011 and 2016 data. This suggests that the differences between the ecological niches for the subtypes were not the result of variability in the measured environmental conditions in the years when each subtype emerged but are driven either by unmeasured environmental variables or by viral differences such as replication rates.

### H5N1 host diffusion within Egypt

A highly supported H5N1 clade (posterior probability = 1.0) within the H5 HA BEAST analysis was identified to investigate viral diffusion among avian hosts in Egypt. This clade was composed of 513 H5N1 HA sequences collected between 2005 and 2017. The vast majority of these sequences (92.4%) were collected from Egyptian hosts. Since chickens were overwhelmingly represented within this sample (67.6%), subsampling was performed to help prevent bias within the model. The resulting sample contained sequences collected from chickens (54), ducks (45), geese (8), turkeys (14), other hosts (6; quail – 3, crow – 1, pigeon – 1 and peacock – 1), and hosts outside of Egypt [19].

**Figure 7.**
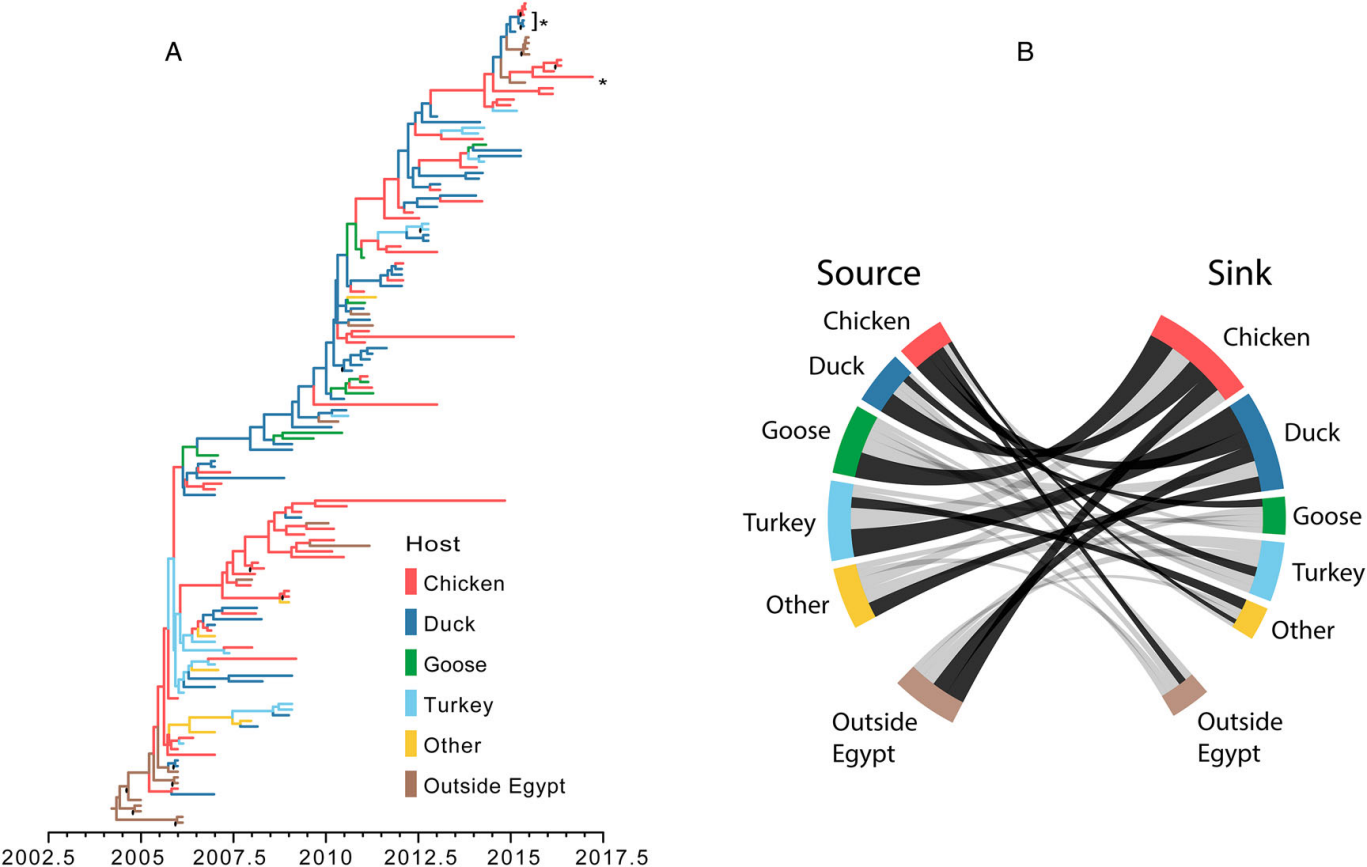
(A) Maximum clade credibility tree of the H5N1 avian host ancestral reconstruction, 2005–2017. Tree branches are coloured based on the highest supported avian host for the descendant node. Nodes with >0.95 posterior support for the ancestral reconstruction are indicated with a black circle (•). Asterisks (∗) indicate sequences collected for this study. (B) Fully resolved discrete trait diffusion network among H5N1 Egyptian hosts. Bands represent transition rates from a source host category (left) to a destination host category (right). The width of the band is proportional to the median transition rate. Dark bands are statistically supported (Bayes factor > 3 and posterior probability > 0.5).

The ancestral reconstruction conveyed a large amount of uncertainty in the host transition history within Egypt (Figure 7(A)). Only 13 ancestral nodes had overwhelming support (posterior probability > 0.95) for a single host. The discrete trait model provided evidence of support for 12 transition rates (Figure 7(B), Table S4). The most frequent transition occurred from turkeys to ducks with a median transition rate of 1.73 transitions per year (95% highest posterior density (HPD) 0.0–3.83; BF = 13.87; pp = 0.76). The transition rate with the highest support occurred from ducks to chickens with a mean transition rate of 1.31 transitions per year (95% HPD 0.13–2.89) and BF = 29.92 (pp = 0.88). Chickens and ducks were involved as either viral sources or sinks in 6 supported transition rates each, suggesting these hosts play substantial roles in the dispersal of H5N1 within Egypt. Furthermore, chickens and ducks were the only hosts with supported transition rates into Egypt (outside Egypt to Egyptian chickens: 1.15 transitions per year, 95% HPD 0.01–2.86, BF = 6.65, pp = 0.61; outside Egypt to Egyptian ducks: 1.00 transitions per year, 95% HPD 0.00–2.77, BF = 4.32, pp = 0.50) as well as migration out of Egypt (Egyptian chickens to outside Egypt: 0.48 transitions per year, 95% HPD 0.01–1.14, BF = 6.20, pp = 0.59).

Because discrete trait diffusion models can be subject to sampling bias, a sensitivity analysis was performed in which the host tip assignments were allowed to be randomized throughout the Bayesian simulation process. When the tips were randomized, root state probabilities (i.e. the posterior probability that the most recent common ancestor existed within a particular host) converged on the prior expectation of a randomized dataset (that is, all hosts have an equal probability of being the root host) (Table S5). Chickens, the most commonly sampled host, had the highest randomized root state probability at 0.27, suggesting sample size may influence the ancestral reconstruction. To further assess the impact of this bias, the magnitude and probability of inclusion of transition rates were compared between the main analysis and the tip randomization (Figures S4 and S5). Rates did not appear to be overinflated by larger sample sizes within the tip randomization. Furthermore, only a single rate estimated under the randomization method reached the level of statistical support (Turkey to Non-Egypt, BF = 3.02). This suggests that while sampling bias may be present within the analysis, it is not enough to significantly influence rate or statistical estimation.

## Discussion

Continuous active surveillance for AIVs in poultry is critical to optimize control and monitor the genesis and emergence of novel strains. Through our systematic active surveillance of AIV in Egypt, four major events were observed: emergence of the H5N8 virus in domestic poultry in Egypt, spread of reassortant H9N2 virus that contains four internal genes from Eurasian viruses, increased detection rate of AIV since the emergence of H5N8, and decreased detection of H5N1 viruses.

During January 2016 to December 2018, we detected an infection rate of 2.9%, with the most commonly isolated subtype being H9N2. During our previous active surveillance in domestic poultry in Egypt, both H5N1 and H9N2 subtypes were commonly detected in birds, with a 5% infection rate (exclusively H5N1 subtype) during August 2009 to July 2010 that increased to 10% (H5N1, H9N2 and H5N1/H9N2 co-infection) from August 2010 to January 2013 [11]. The detection rate decreased to 4.6% (H5N1, H9N2 and H5N1/H9N2 co-infection) during February 2013 to December 2015 with no detection of H5N1 viruses in the last quarter of 2015 [17].

We have previously shown that the H9N2 virus was becoming more prevalent in Egypt than H5N1 as of the last quarter of 2015 [17]. The current study data indicates that H9N2 and H5N8 are more prevalent than H5N1 whose detection is becoming a rare occurrence. The ENM was conducted to understand whether those subtypes favour specific geographical niches. Results supported our surveillance findings and indicated that although H5N1 favours a wide geographic region, it competes with H9N2 in the Nile Delta region and with H5N8 in middle and southern Egypt. We hypothesized that H5N1 might be losing this competition due to viral factors such as replication rates or other unmeasured factors. H5N1 were found to have the lowest replication efficiency when compared to H9N2 and H5N8 viruses. Another report also noted that H5N8 viruses had a high replication rate giving HA titres of 10–10.5 log_2_ [42]. Therefore, we predict that H9N2 and H5N8 will become the main circulating viruses in Egyptian poultry as both grow more efficiently than H5N1 and prefer the same ecological niches. H9N2 will likely be more prevalent in the Nile Delta region where most of the poultry production occurs while H5N8 will be more common in Fayoum and other areas to the south of the delta. It is recommended that Egyptian veterinary authorities revise their prevention and control measures in light of those findings only after the replication efficacies of other viruses are tested to corroborate our results.

Although H5N1 prevalence has declined, investigation of historical host transitions reveals important information regarding AIV control and biosecurity within Egypt. Domestic chickens and ducks appear to play a key role not only in the dispersal of H5N1 within Egypt, but also in migrations of the virus into and out of the country, consistent with a hypothesis of trade driven viral spread [43]. While oversampling of these hosts may influence the results, our sensitivity analysis suggests that sampling bias does not significantly impact statistical estimation. In addition, because chickens and ducks are the most commonly reared birds in Egypt and tend to have higher prevalence of AIV, their larger proportions within the model are justified. Unfortunately, livestock production type (i.e. backyard vs. commercial production) was not included within the diffusion model, limiting the ability of the model to evaluate the role of agricultural methods on inter-species viral transmission. Further sequencing and analysis of H9N2 and H5N8 isolates will be required to investigate whether H5N1 host transition patterns can be generalized to other subtypes within Egypt.

Analysis of the H5N1 sequences in this study did not indicate novel phylogenetic patterns. The HA sequences of the newly-isolated H5N1 viruses were closely related to previously characterized clade 2.2.1.2 viruses [4]. Two antigenically distinguishable groups of H5N1 were characterized. The first group included only A/chicken/Egypt/B13826D/2017(H5N1) virus that drifted as a result of losing a glycosylation site at position 23 and carrying three mutations E127D, R140K (antigenic site A), and T202A in the HA glycoprotein. The second group had several H5N1 strains from 2013 and 2014, that shared the R140K mutation in the antigenic site A of the HA. The HA glycosylation sites are well known as important means for the evolution of influenza A viruses [44].

In previous studies, we found no evidence that the H9N2 isolates from 2011 to 2015 were undergoing antigenic drift except for isolates from quail [10,45]; the same observation was made with the more recent viruses. Although vaccination with inactivated vaccines containing H9N2 antigens originating from Egypt or elsewhere in Asia has been used in Egypt since 2012, the campaign was not well-implemented potentially accelerating antigenic drift and vaccine escape as seen with the H5N1 viruses [46]. There is, however, limited evidence for this with H9N2 isolates from chickens, pigeons, and ducks remaining antigenically conserved with distinct viruses only seen in quail [10,45].

Genetic analyses of all eight genes of 4 H9N2 viruses characterized in this study revealed reassortment events in internal genes of PB2, PB1, PA and NS of Egyptian H9N2 strains with Eurasian AIVs circulating in wild birds. Our previous study indicated H9N2 virus isolated from domestic pigeons in 2014 inherited five internal genes (PB2, PB1, PA, NP, and NS) from Eurasian AIVs [14].

Counter to the previous study that characterized several genetically distinct influenza A(H5N8) viruses isolated from different governorates [47], full genomes of H5N8 viruses generated in this study were identical.

Selection bias may have affected the results shown here as not all geographic areas of Egypt were sampled and convenience sampling was used. However, the main aim of this work was to study the genetic and antigenic characteristics of the viruses circulating at the time of the study. Hence, our detection rates may not indicate the true incidence or prevalence of AI infection among Egyptian poultry. Another issue is the lack of data on vaccinations used in the sampled sites. If vaccines are heavily used, then the estimates provided in this study will be underestimated and the analysis by bird health can be biased.

In Egypt, co-circulation of HPAI H5N1 and H5N8 with H9N2 viruses among poultry has been observed. Eradication of these viruses from Egypt is considered unlikely due to gaps in the application of recommended AIV control strategies. Global active surveillance of AIV among domestic and migratory wild birds needs to be sustained to monitor the spread and genesis of circulating viruses.

## Supplementary Material

Supplemental Material
