# Supplementary material for: Active surveillance and genetic evolution of avian influenza viruses in Egypt, 2016–2018
Source: Emerg Microbes Infect. 2019 Sep 17;8(1):1370–82. doi: 10.1080/22221751.2019.1663712 (PMC6758608; doi:10.1080/22221751.2019.1663712)
Supplement: Supplemental Material [file TEMI_A_1663712_SM3456.zip › Supplement_Table_S1.docx]

**Table S1**: Median nucleotide substitution rates of HA genes among represented AIV subtypes.

| **Subtype** | **Median** | **95% HPD – Lower** | **95% HPD – Upper** |
| --- | --- | --- | --- |
| H5N1 | 4.39E-03 | 3.85E-03 | 4.93E-03 |
| H5N8 | 3.63E-03 | 2.58E-03 | 5.08E-03 |
| N9N2 | 6.52E-03 | 5.69E-03 | 7.37E-03 |

95% HPD – 95% highest posterior density Bayesian credibility interval
