# Supplementary material for: Active surveillance and genetic evolution of avian influenza viruses in Egypt, 2016–2018
Source: Emerg Microbes Infect. 2019 Sep 17;8(1):1370–82. doi: 10.1080/22221751.2019.1663712 (PMC6758608; doi:10.1080/22221751.2019.1663712)
Supplement: Supplemental Material [file TEMI_A_1663712_SM3456.zip › Supplement_Table_S2.docx]

| **Table S2**: Strain names with GenBank accession numbers for sequences generated in this study | **Accession number** | | | | | | | |
| --- | --- | --- | --- | --- | --- | --- | --- | --- |
| **Isolate** | **PB2** | **PB1** | **PA** | **HA** | **NP** | **NA** | **M** | **NS** |
| **A/pigeon/Egypt/A15052/2018 (H5N8)** |  |  |  | MN038184 |  |  |  |  |
| **A/chicken/Egypt/F15099/2018 (H5N8)** |  |  |  | MN038194 |  |  |  |  |
| **A/chicken/Egypt/N15173D/2018(H5N8)** |  |  |  | MN038195 |  |  |  |  |
| **A/duck/Egypt/N13736E/2017 (H5N8)** | MH498579 | MH498560 | MH498616 | MH498625 | MH498576 | MH498591 | MH498583 | MH498555 |
| **A/duck/Egypt/F13667A/2017 (H5N8)** |  |  |  | MK190702 |  | MK190703 |  |  |
| **A/duck/Egypt/F13666A/2017 (H5N8)** | MH498566 | MH498601 | MH498580 | MH498622 | MH498619 | MH498574 | MH498609 | MH498564 |
| **A/chicken/Egypt/F13660A/2017 (H5N8)** |  |  |  | MH998478 |  | MH998479 |  |  |
| **A/duck/Egypt/F13663C/2017 (H5N8)** |  |  |  | MK190697 |  | MK190698 |  |  |
| **A/chicken/Egypt/Q13804A/2017 (H5N8)** | MH498557 | MH498582 | MH498559 | MH498577 | MH498603 | MH498607 | MH498592 | MH498628 |
| **A/chicken/Egypt/F13664A/201 (H5N8)** |  |  |  | MH998490 |  | MH998491 |  |  |
| **A/chicken/Egypt/A15068/2018 (H9N2)** |  |  |  | MN038183 |  |  |  |  |
| **A/chicken/Egypt/A15074/2018 (H9N2)** |  |  |  | MN038193 |  |  |  |  |
| **A/chicken/Egypt/D13646A/2017 (H9N2)** | MH498627 | MH498597 | MH498570 | MH498586 | MH498593 | MH498620 | MH498584 | MH498554 |
| **A/chicken/Egypt/S12568C/2016 (H9N2)** | MK190710 | MK190706 | MK190709 | KY558857 | MK190707 | KY558854 | MK190708 | MK190705 |
| **A/quail/Egypt/S13424/2016 (H9N2)** | MK007983 | MK007986 | MK007989 | MK007990 | MK007985 | MK007988 | MK007984 | MK007987 |
| **A/pigeon/Egypt/S11755/2015 (H9N2)** | MK007977 | MK007975 | MK007980 | MK007981 | MK007982 | MK007979 | MK007978 | MK007976 |
| **A/chicken/Egypt/N12643B/2016 (H5N1)** |  |  |  | KY558850 |  | KY558869 |  |  |
| **A/chicken/Egypt/N12638D/2016 (H5N1)** |  |  |  | KY558870 |  | KY558849 |  |  |
| **A/chicken/Egypt/N12640A/2016 (H5N1)** |  |  |  | KY558858 |  | KY558843 |  |  |
| **A/chicken/Egypt/N12642E/2016 (H5N1)** |  |  |  | KY558852 |  | KY558862 |  |  |
| **A/chicken/Egypt/B13825A/2017 (H5N1)** | MH498575 | MH498590 | MH498611 | MH498571 | MH498578 | MH498569 | MH498624 | MH498631 |
| **A/chicken/Egypt/F12505E/2016 (H5N1)** | MH513604 | MH513607 | MH513603 | KY558844 | MH513602 | KY558863 | MH513606 | MH513605 |
| **A/chicken/Egypt/H11100D/2015 (H5N1)** |  |  |  | MK190711 |  |  |  |  |
| **A/chicken/Egypt/H11100E/2015 (H5N1)** |  |  |  | MK190700 |  |  |  |  |
| **A/chicken/Egypt/H11111B/2015 (H5N1)** |  |  |  | MK190692 |  |  |  |  |
| **A/chicken/Egypt/A11111C/2015 (H5N1)** |  |  |  | MK190701 |  |  |  |  |
| **A/chicken/Egypt/H11098A/2015 (H5N1)** |  |  |  | MK190696 |  |  |  |  |
| **A/chicken/Egypt/H11098C/2015 (H5N1)** |  |  |  | MK190719 |  |  |  |  |
| **A/chicken/Egypt/H11098D/2015 (H5N1)** |  |  |  | MK190724 |  |  |  |  |
| **A/duck/Egypt/H11025A/2015 (H5N1)** |  |  |  | MK190725 |  |  |  |  |
| **A/duck/Egypt/H11025B/2015 (H5N1)** |  |  |  | MK190733 |  |  |  |  |
| **A/duck/Egypt/H11025D/2015 (H5N1)** |  |  |  | MK190731 |  |  |  |  |
| **A/duck/Egypt/H11025E/2015 (H5N1)** |  |  |  | MK190732 |  |  |  |  |
| **A/chicken/Egypt/H11029/2015 (H5N1)** |  |  |  | MK190735 |  |  |  |  |
| **A/chicken/Egypt/H11033/2015 (H5N1)** |  |  |  | MK190734 |  |  |  |  |
| **A/chicken/Egypt/H11034/2015 (H5N1)** |  |  |  | MK190720 |  |  |  |  |
| **A/chicken/Egypt/H11035/2015 (H5N1)** |  |  |  | MK190704 |  |  |  |  |
| **A/chicken/Egypt/H11036/2015 (H5N1)** |  |  |  | MK190718 |  |  |  |  |
| **A/chicken/Egypt/H11037/2015 (H5N1)** |  |  |  | MK190723 |  |  |  |  |
| **A/chicken/Egypt/H11038/2015 (H5N1)** |  |  |  | MK190721 |  |  |  |  |
| **A/chicken/Egypt/H11098E/2015 (H5N1)** |  |  |  | MK190726 |  |  |  |  |
| **A/chicken/Egypt/H11099A/2015 (H5N1)** |  |  |  | MK190699 |  |  |  |  |
| **A/chicken/Egypt/H11099B/2015 (H5N1)** |  |  |  | MK190722 |  |  |  |  |
| **A/chicken/Egypt/H11099C/2015 (H5N1)** |  |  |  | MK190693 |  |  |  |  |
| **A/chicken/Egypt/H11100A/2015 (H5N1)** |  |  |  | MK190694 |  |  |  |  |
| **A/chicken/Egypt/H11100C/2015 (H5N1)** |  |  |  | MK190691 |  |  |  |  |
