# Supplementary material for: Active surveillance and genetic evolution of avian influenza viruses in Egypt, 2016–2018
Source: Emerg Microbes Infect. 2019 Sep 17;8(1):1370–82. doi: 10.1080/22221751.2019.1663712 (PMC6758608; doi:10.1080/22221751.2019.1663712)
Supplement: Supplemental Material [file TEMI_A_1663712_SM3456.zip › Supplement_Table_S3.docx]

**Table S3A:** Genetic markers in the HA genes of H5 viruses.

| Virus | Receptor binding sites | | HA Cleavage site | Antigenic site A | | | | Antigenic site B | | | Antigenic site E | | | New antigeic site | | | | | | | N-linked glycosylation site | | | | | |
| --- | --- | --- | --- | --- | --- | --- | --- | --- | --- | --- | --- | --- | --- | --- | --- | --- | --- | --- | --- | --- | --- | --- | --- | --- | --- | --- |
|  | 222 | 224 | 321 | 127 | 133 | 140 | 141 | 154 | 156 | 184 | 71 | 83 | 86 | 94 | 120 | 162 | 227 | 252 | 263 | 282 | 10 | 23 | 165 | 286 | 483 | 542 |
| Clade 2.2.1.2 (Egyptian H5N1) | Q | G | PQGEKRRKKR/PQGERRRKKR/PQGGKRRKKR | E | S | R | S/P | N/S | A/S | A | L/R | I/N | A/T/V | N | D | K | E | N | T/S | I | NNST | NVTV | NNTN | NSSM | NGTY | NGSL |
| A/chicken/Egypt/H11098E/2015 (H5N1) | . | . | PQGEKRRKKR | . | . | . | S | N | A | . | L | I | A | . | D | . | . | . | T | . | …. | …. | …. | …. | …. | …. |
| A/chicken/Egypt/H11099A/2015 (H5N1) | . | . | PQGEKRRKKR | . | . | . | S | N | A | . | L | I | A | . | D | . | . | . | T | . | …. | …. | …. | …. | …. | …. |
| A/chicken/Egypt/H11099B/2015 (H5N1) | . | . | PQGEKRRKKR | . | . | . | S | N | A | . | L | I | A | . | D | . | . | . | T | . | …. | …. | …. | …. | …. | …. |
| A/chicken/Egypt/H11099C/2015 (H5N1) | . | . | PQGEKRRKKR | . | . | . | S | N | A | . | L | I | A | . | D | . | . | . | T | . | …. | …. | …. | …. | …. | …. |
| A/chicken/Egypt/H11100A/2015 (H5N1) | . | . | PQGEKRRKKR | . | . | . | S | N | A | . | L | I | A | . | D | . | . | . | T | . | …. | …. | …. | …. | …. | …. |
| A/chicken/Egypt/H11100C/2015 (H5N1) | . | . | PQGEKRRKKR | . | . | . | S | N | A | . | L | I | A | . | D | . | . | . | T | . | …. | …. | …. | …. | …. | …. |
| A/chicken/Egypt/H11100D/2015 (H5N1) | . | . | PQGEKRRKKR | . | . | . | S | N | A | . | L | I | A | . | D | . | . | . | T | . | …. | …. | …. | …. | …. | …. |
| A/chicken/Egypt/H11100E/2015 (H5N1) | . | . | PQGEKRRKKR | . | . | . | S | N | A | . | L | I | A | . | D | . | . | . | T | . | …. | …. | …. | …. | …. | …. |
| A/chicken/Egypt/H11111B/2015 (H5N1) | . | . | PQGEKRRKKR | . | . | . | S | N | A | . | L | I | A | . | D | . | . | . | T | . | …. | …. | …. | …. | …. | …. |
| A/chicken/Egypt/H11111C/2015 (H5N1) | . | . | PQGEKRRKKR | . | . | . | S | N | A | . | L | I | A | . | D | . | . | . | T | . | …. | …. | …. | …. | …. | …. |
| A/duck/Egypt/H11025A/2015 (H5N1) | . | . | PQGEKRRKKR | . | . | . | S | N | A | . | L | I | A | . | D | . | . | . | T | . | …. | …. | …. | …. | …. | …. |
| A/duck/Egypt/H11025B/2015 (H5N1) | . | . | PQGEKRRKKR | . | . | . | S | N | A | . | L | I | A | . | D | . | . | . | T | . | …. | …. | …. | …. | …. | …. |
| A/duck/Egypt/H11025D/2015 (H5N1) | . | . | PQGEKRRKKR | . | . | . | S | N | A | . | L | I | A | . | D | . | . | . | T | . | …. | …. | …. | …. | …. | …. |
| A/duck/Egypt/H11025E/2015 (H5N1) | . | . | PQGEKRRKKR | . | . | . | S | N | A | . | L | I | A | . | D | . | . | . | T | . | …. | …. | …. | …. | …. | …. |
| A/chicken/Egypt/H11029/2015 (H5N1) | . | . | PQGEKRRKKR | . | . | . | S | N | A | . | L | I | A | . | D | . | . | . | T | . | …. | …. | …. | …. | …. | …. |
| A/chicken/Egypt/H11033/2015 (H5N1) | . | . | PQGEKRRKKR | . | . | . | S | N | A | . | L | I | A | . | D | . | . | . | T | . | …. | …. | …. | …. | …. | …. |
| A/chicken/Egypt/H11034/2015 (H5N1) | . | . | PQGEKRRKKR | . | . | . | S | N | A | . | L | I | A | . | D | . | . | . | T | . | …. | …. | …. | …. | …. | …. |
| A/chicken/Egypt/H11035/2015 (H5N1) | . | . | PQGEKRRKKR | . | . | . | S | N | A | . | L | I | A | . | D | . | . | . | T | . | …. | …. | …. | …. | …. | …. |
| A/chicken/Egypt/H11036/2015 (H5N1) | . | . | PQGEKRRKKR | . | . | . | S | N | A | . | L | I | A | . | D | . | . | . | T | . | …. | …. | …. | …. | …. | …. |
| A/chicken/Egypt/H11037/2015 (H5N1) | . | . | PQGEKRRKKR | . | . | . | S | N | A | . | L | I | A | . | D | . | . | . | T | . | …. | …. | …. | …. | …. | …. |
| A/chicken/Egypt/H11038/2015 (H5N1) | . | . | PQGEKRRKKR | . | . | . | S | N | A | . | L | I | A | . | D | . | . | . | T | . | …. | …. | …. | …. | …. | …. |
| A/chicken/Egypt/H11098A/2015 (H5N1) | . | . | PQGEKRRKKR | . | . | . | S | N | A | . | L | I | A | . | D | . | . | . | T | . | …. | …. | …. | …. | …. | …. |
| A/chicken/Egypt/H11098C/2015 (H5N1) | . | . | PQGEKRRKKR | . | . | . | S | N | A | . | L | I | A | . | D | . | . | . | T | . | …. | …. | …. | …. | …. | …. |
| A/chicken/Egypt/H11098D/2015 (H5N1) | . | . | PQGEKRRKKR | . | . | . | S | N | A | . | L | I | A | . | D | . | . | . | T | . | …. | …. | …. | …. | …. | …. |
| A/Chicken/Egypt/B13825A/2017 (H5N1) | . | . | PQGEKRRKKR | D | . | K | S | N | A | . | L | I | A | . | E | . | . | . | T | . | …. | …. | …. | …. | …. | …. |
| A/chciken/Egypt/N12642E/2016 (H5N1) | . | . | PQGEKRRKKR | . | . | . | S | N | A | . | L | I | A | . | E | . | . | . | T | . | …. | …. | …. | …. | …. | …. |
| A/chciken/Egypt/N12643B/2016 (H5N1) | . | . | PQGEKRRKKR | . | . | . | S | N | A | . | L | I | A | . | E | . | . | . | T | . | …. | …. | …. | …. | …. | …. |
| A/chciken/Egypt/F12505B/2016 (H5N1) | . | . | PQGEKRRKKR | . | . | . | S | N | A | . | L | I | A | . | G | . | . | . | T | . | …. | …. | …. | …. | …. | …. |
| A/chciken/Egypt/F12505C/2016 (H5N1) | . | . | PQGEKRRKKR | . | . | . | S | N | A | . | L | I | A | . | G | . | . | . | T | . | …. | …. | …. | …. | …. | …. |
| A/chciken/Egypt/N12638D/2016 (H5N1) | . | . | PQGEKRRKKR | . | . | . | S | N | A | . | L | I | A | . | E | . | . | . | T | . | …. | …. | …. | …. | …. | …. |
| A/chicken/Egypt/N12640A/2016 (H5N1) | . | . | PQGEKRRKKR | . | . | . | S | N | A | . | L | I | A | . | E | . | . | . | T | . | …. | …. | …. | …. | …. | …. |
| A/chicken/Egypt/F12505E/2016 (H5N1) | . | . | PQGEKRRKKR | . | . | . | S | N | A | . | L | I | A | . | G | . | . | . | T | . | …. | …. | …. | …. | …. | …. |
| A/chicken/Egypt/B13826D/2017(H5N1) | . | . | PQGEKRRKKR | D | . | K | S | N | A | . | L | I | A | . | E | . | . | . | T | . | …. | SVTV | …. | …. | …. | …. |
| A/duck/Egypt/F13667A/2017 (H5N8) | . | . | PLREKRRKR | . | A | T | P | N | A | A | L | A | A | S | S | I | D | Y | T | V | …. | …. | …. | …. | …. | …. |
| A/chicken/Egypt/F13660A/2017 (H5N8) | . | . | PLREKRRKR | . | A | T | P | N | E | A | L | A | A | S | S | I | D | Y | T | V | …. | …. | …. | …. | …. | …. |
| A/duck/Egypt/F13663C/2017 (H5N8) | . | . | PLREKRRKR | . | A | T | P | N | A | A | L | A | A | S | S | I | D | Y | T | V | …. | …. | …. | …. | …. | …. |
| A/duck/Egypt/F13666A/2017 (H5N8) | . | . | PLREKRRKR | . | A | T | P | N | A | A | L | A | A | S | S | I | D | Y | T | V | …. | …. | …. | …. | …. | …. |
| A/pigeon/Egypt/A15052/2018 (H5N8) | . | . | PLREKRRKR | . | A | A | P | N | A | A | L | A | A | S | S | I | D | Y | T | V | …. | …. | …. | …. | …. | …. |
| A/chicken/Egypt/F15099/2018 (H5N8) | . | . | PLREKRRKR | . | A | T | P | N | A | A | L | A | A | S | S | I | D | Y | T | V | …. | …. | …. | …. | …. | …. |
| A/chicken/Egypt/N15173D/2018(H5N8) | . | . | PLREKRRKR | . | A | T | P | N | A | A | L | A | A | S | S | I | D | Y | T | V | …. | …. | …. | …. | …. | …. |
| A/duck/Egypt/N13736E/2017 (H5N8) | . | . | PLREKRRKR | . | A | T | P | N | A | A | L | A | A | S | S | I | D | Y | T | V | …. | …. | …. | …. | …. | …. |
| A/chicken/Egypt/Q13804A/2017 (H5N8) | . | . | PLREKRRKR | . | A | T | P | N | A | A | L | A | A | S | S | I | D | Y | T | V | …. | …. | …. | …. | …. | …. |

**Table S3B:** Genetic markers in the HA genes of H9 viruses.

| **H9N2 Virus** | **Receptor binding sites** | | | | | | | | **Glycosylation sites** | | | | | | | |  | **Antigenic Site I (A)** | | | **Antigenic Site II (B)** | | | **Overlapping site** | | |
| --- | --- | --- | --- | --- | --- | --- | --- | --- | --- | --- | --- | --- | --- | --- | --- | --- | --- | --- | --- | --- | --- | --- | --- | --- | --- | --- |
|  |  |  |  |  |  |  |  |  |  |  |  |  |  |  |  |  |  | **143** | **166** | **170** | **153** | **201** | **234** | **141** | **197** | **206** |
|  | **166** | **191** | **197** | **198** | **232** | **234** | **235** | **236** | **29** | **105** | **141** | **196** | **206** | **218** | **298** | **305** | **492** | T | N | P | D | N | L | N | T | T |
| **A/quail/Egypt/D10093/2014** | **D** | **H** | **T** | **T** | **N** | **L** | **T** | **G** | NST | NGT | NVT | **NTT** | **TDT** | **DRT** | NST | NIS | NGT | . | D | . | N | A | . | . | . | . |
| **A/quail/Egypt/D10105/2014** | **D** | **.** | **.** | **T** | **.** | **.** | **T** | **.** | **. . .** | **. . .** | **. . .** | **N.T** | **T . .** | **D . .** | **. . .** | **. . .** | **. . .** | . | D | . | N | A | . | . | . | . |
| **A/ quail /Egypt/D10106/2014** | **D** | **.** | **.** | **T** | **.** | **.** | **T** | **.** | **. . .** | **. . .** | **. . .** | **N.T** | **T . .** | **D . .** | **. . .** | **. . .** | **. . .** | . | D | . | N | A | . | . | . | . |
| **A/chicken/F10285D/2014** | **N** | **.** | **.** | **A** | **.** | **.** | **I** | **.** | **. . .** | **. . .** | **. . .** | **D.A** | **T . .** | **D . .** | **. . .** | **. . .** | **. . .** | . | . | . | G | . | . | . | . | . |
| **A/chicken/Egypt/F10993B/2015** | **N** | **.** | **.** | **A** | **.** | **.** | **I** | **.** | . . . | . . . | . . . | D.A | T . . | D . . | . . . | . . . | . . . | . | . | . | . | . | . | . | . | . |
| **A/chicken/Egypt/S12568C/2016** | **N** | **.** | **.** | A | **.** | **.** | **I** | **.** | . . . | . . . | . . . | D.A | T . . | D . . | . . . | . . . | . . . | . | . | . | . | . | . | . | . | . |
| **A/chicken/Egypt/F12168D/2016** | **N** | **.** | **.** | A | **.** | **.** | **I** | **.** | . . . | . . . | . . . | D.A | T . . | D . . | . . . | . . . | . . . | . | . | . | . | . | . | . | . | . |
| **A/chicken/Egypt/F12170A/2016** | **N** | **.** | **.** | T | **.** | **.** | **I** | **.** | . . . | . . . | . . . | D.T | T . . | D . . | . . . | . . . | . . . | . | . | . | . | . | . | . | . | . |
| **A/chicken/Egypt/F12168B/2016** | **N** | **.** | **.** | A | **.** | **.** | **I** | **.** | . . . | . . . | . . . | D.A | T . . | D . . | . . . | . . . | . . . | . | . | . | . | . | . | . | . | . |
| **A/chicken/Egypt/S12568C/2016** | **N** | **.** | **.** | A | **.** | **.** | **I** | **.** | . . . | . . . | . . . | D.A | T . . | D . . | . . . | . . . | . . . | . | . | . | . | . | . | . | . | . |
| **A/chicken/Egypt/F12170E/2016** | **N** | **.** | **.** | T | **.** | **.** | **I** | **.** | . . . | . . . | . . . | D.T | T . . | D . . | . . . | . . . | . . . | . | . | . | . | . | . | . | . | . |
| **A/chicken/Egypt/F12173D/2016** | **N** | **.** | **.** | A | **.** | **.** | **I** | **.** | . . . | . . . | . . . | D.A | T . . | D . . | . . . | . . . | . . . | . | . | . | . | . | . | . | . | . |
| **A/chicken/Egypt/F12054D/2016** | **N** | **.** | **.** | A | **.** | **.** | **I** | **.** | . . . | . . . | . . . | D.A | T . . | D . . | . . . | . . . | . . . | . | . | . | . | . | . | . | . | . |
| **A/chicken/Egypt/D13646A/2017** | **N** | **.** | **.** | A | **.** | **.** | **I** | **.** | . . . | . . . | . . . | D.A | T . . | D . . | . . . | . . . | . . . | . | . | . | . | . | . | . | . | . |
| **A/chicken/Egypt/A15068/2018** | **N** | **.** | **.** | A | **.** | **.** | **I** | **.** | . . . | . . . | . . . | D.A | T . . | D . . | . . . | . . . | . . . | . | . | . | . | . | . | . | . | . |
| **A/chicken/Egypt/A15074/2018** | **N** | **.** | **.** | A | **.** | **.** | **I** | **.** | . . . | . . . | . . . | D.A | T . . | D . . | . . . | .V. | . . . | . | . | . | . | . | . | . | . | . |
