# Supplementary material for: Active surveillance and genetic evolution of avian influenza viruses in Egypt, 2016–2018
Source: Emerg Microbes Infect. 2019 Sep 17;8(1):1370–82. doi: 10.1080/22221751.2019.1663712 (PMC6758608; doi:10.1080/22221751.2019.1663712)
Supplement: Supplemental Material [file TEMI_A_1663712_SM3456.zip › Supplement_Table_S4.docx]

**Table S4:** Median transition rates of the Egyptian H5N1 host discrete trait diffusion model, 2005–2017. Traits are reported in transitions per year with corresponding 95% highest posterior density (HPD) intervals in brackets, Bayes factors (BF) and posterior probabilities (pp).

| Source | Chicken | Duck | Goose | Turkey | Other | Outside  Egypt |
| --- | --- | --- | --- | --- | --- | --- |
| Chicken |  | 1.02  [0.01, 2.22]  BF = 11.37  pp = 0.73 | 0.36  [0.01, 0.91]  BF = 1.72  pp =0.29 | 0.59  [0.00, 1.37]  BF = 12.12  pp = 0.74 | 0.28  [0.01, 0.72]  BF = 6.15  pp = 0.59 | 0.48  [0.01, 1.14]  BF = 6.20  pp = 0.59 |
| Duck | 1.31  [0.13, 2.89]  BF = 29.92  pp = 0.88 |  | 0.49  [0.02, 1.19]  BF = 9.04  pp = 0.68 | 0.51  [0.00, 1.40]  BF = 1.29  pp = 0.23 | 0.24  [0.00, 0.76]  BF = 0.72  pp = 0.14 | 0.50  [0.00, 1.20]  BF = 4.25  pp = 0.50 |
| Goose | 1.59  [0.00, 3.68]  BF = 12.99  pp = 0.75 | 0.99  [0.00, 2.87]  BF = 2.29  pp = 0.35 |  | 0.58  [0.00, 2.24]  BF = 1.20  pp = 0.22 | 0.35  [0.00, 1.54]  BF = 0.64  pp = 0.13 | 0.46  [0.00, 2.05]  BF = 0.81  pp = 0.16 |
| Turkey | 1.34  [0.00, 3.51]  BF = 4.13  pp = 0.49 | 1.73  [0.00, 3.83]  BF = 13.87  pp = 0.76 | 0.39  [0.00, 1.65]  BF = 0.77  pp = 0.15 |  | 0.67  [0.00, 1.89]  BF = 4.37  pp = 0.51 | 0.40  [0.00, 1.76]  BF = 0.67  pp = 0.14 |
| Other | 0.80  [0.00, 2.65]  BF = 2.08  pp = 0.33 | 0.96  [0.00, 2.93]  BF = 4.53  pp = 0.51 | 0.43  [0.00, 1.99]  BF = 0.89  pp = 0.17 | 0.87  [0.00, 2.61]  BF = 3.39  pp = 0.44 |  | 0.45  [0.00, 2.22]  BF = 0.94  pp = 0.18 |
| Outside  Egypt | 1.15  [0.01, 2.86]  BF = 6.65  pp = 0.61 | 1.00  [0.00, 2.77]  BF = 4.32  pp = 0.50 | 0.47  [0.00, 1.65]  BF = 0.95  pp = 0.18 | 0.82  [0.00, 2.45]  BF = 2.73  pp = 0.39 | 0.36  [0.00, 1.41]  BF = 0.64  pp = 0.13 |  |
