# Supplementary material for: Active surveillance and genetic evolution of avian influenza viruses in Egypt, 2016–2018
Source: Emerg Microbes Infect. 2019 Sep 17;8(1):1370–82. doi: 10.1080/22221751.2019.1663712 (PMC6758608; doi:10.1080/22221751.2019.1663712)
Supplement: Supplemental Material [file TEMI_A_1663712_SM3456.zip › Supplement_Table_S5.docx]

**Table S5:** Comparison of root state probabilities between the main discrete trait diffusion analysis and the tip randomization sensitivity analysis.

| Host | Main Analysis | Tip Randomization |
| --- | --- | --- |
| Goose | 0.0078 | 0.1198 |
| Turkey | 0.0059 | 0.216 |
| Other | 0.006 | 0.14 |
| Non-Egypt | 0.9416 | 0.1215 |
| Duck | 0.0142 | 0.1346 |
| Chicken | 0.0246 | 0.2681 |
