# Supplementary figures and images for: Active surveillance and genetic evolution of avian influenza viruses in Egypt, 2016–2018
Source: Emerg Microbes Infect. 2019 Sep 17;8(1):1370–82. doi: 10.1080/22221751.2019.1663712 (PMC6758608; doi:10.1080/22221751.2019.1663712)

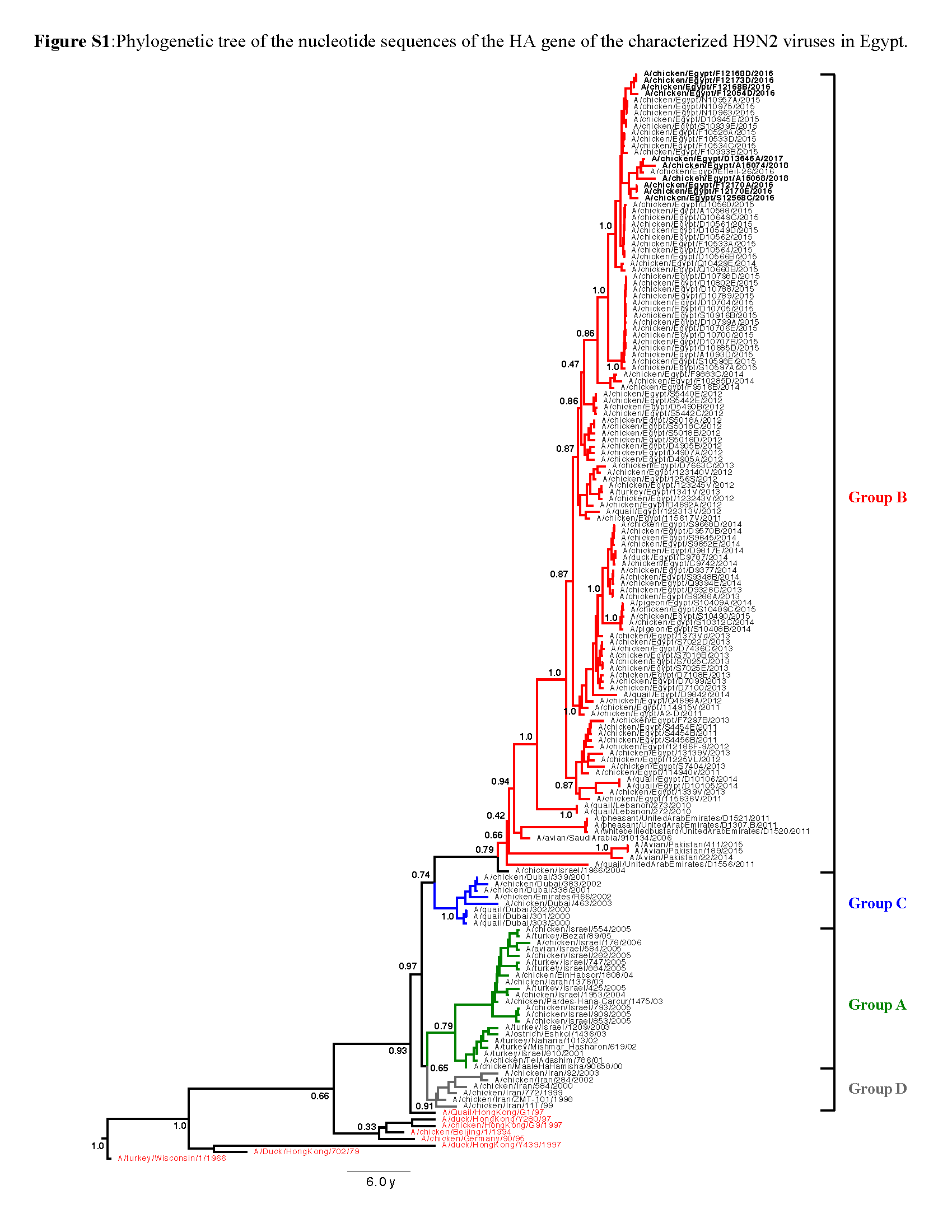

Supplement: Supplemental Material [file TEMI_A_1663712_SM3456.zip › Supplement Figure 1_final.tif]

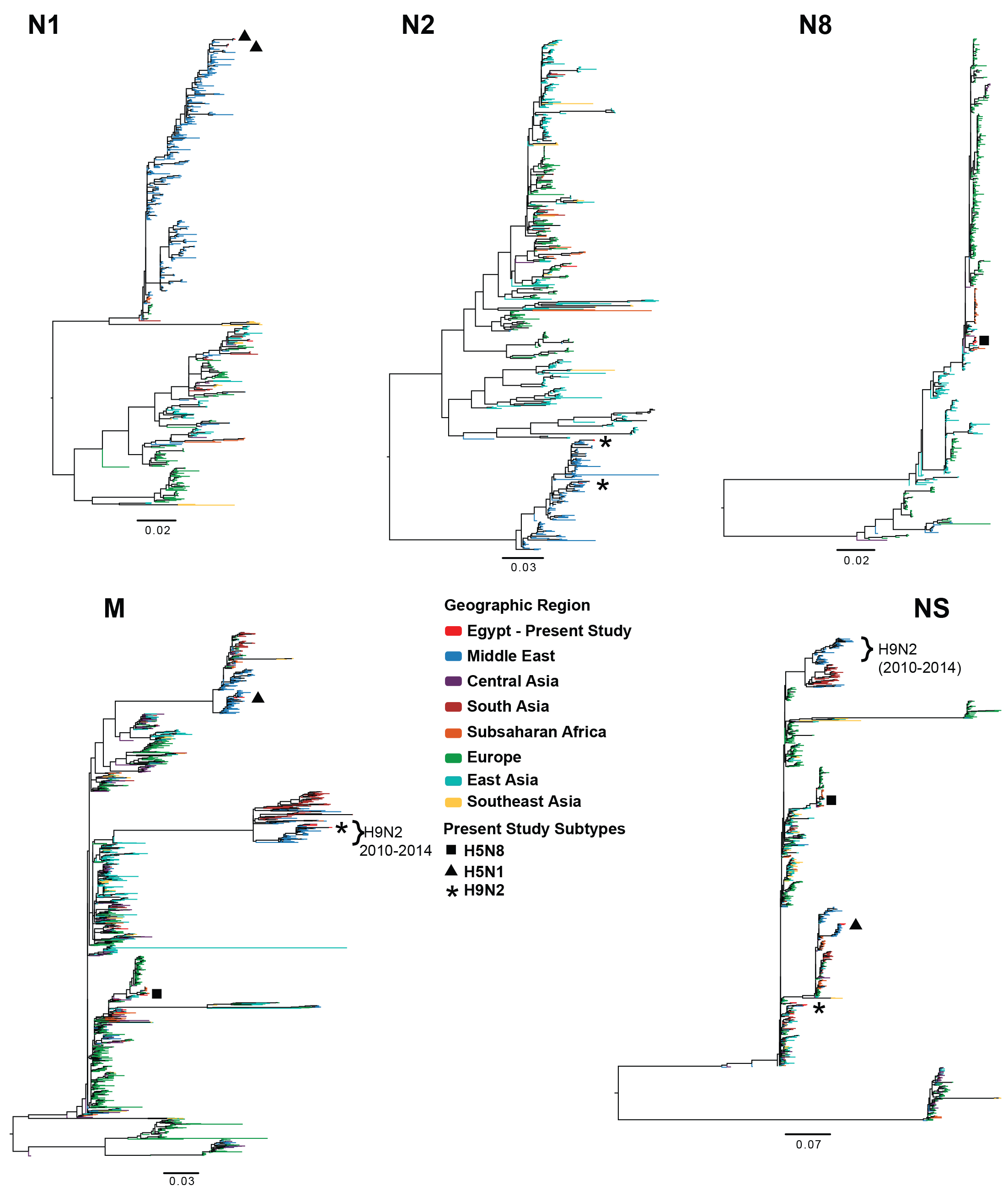

Supplement: Supplemental Material [file TEMI_A_1663712_SM3456.zip › Supplement Figure 2-page 1_final.tif]

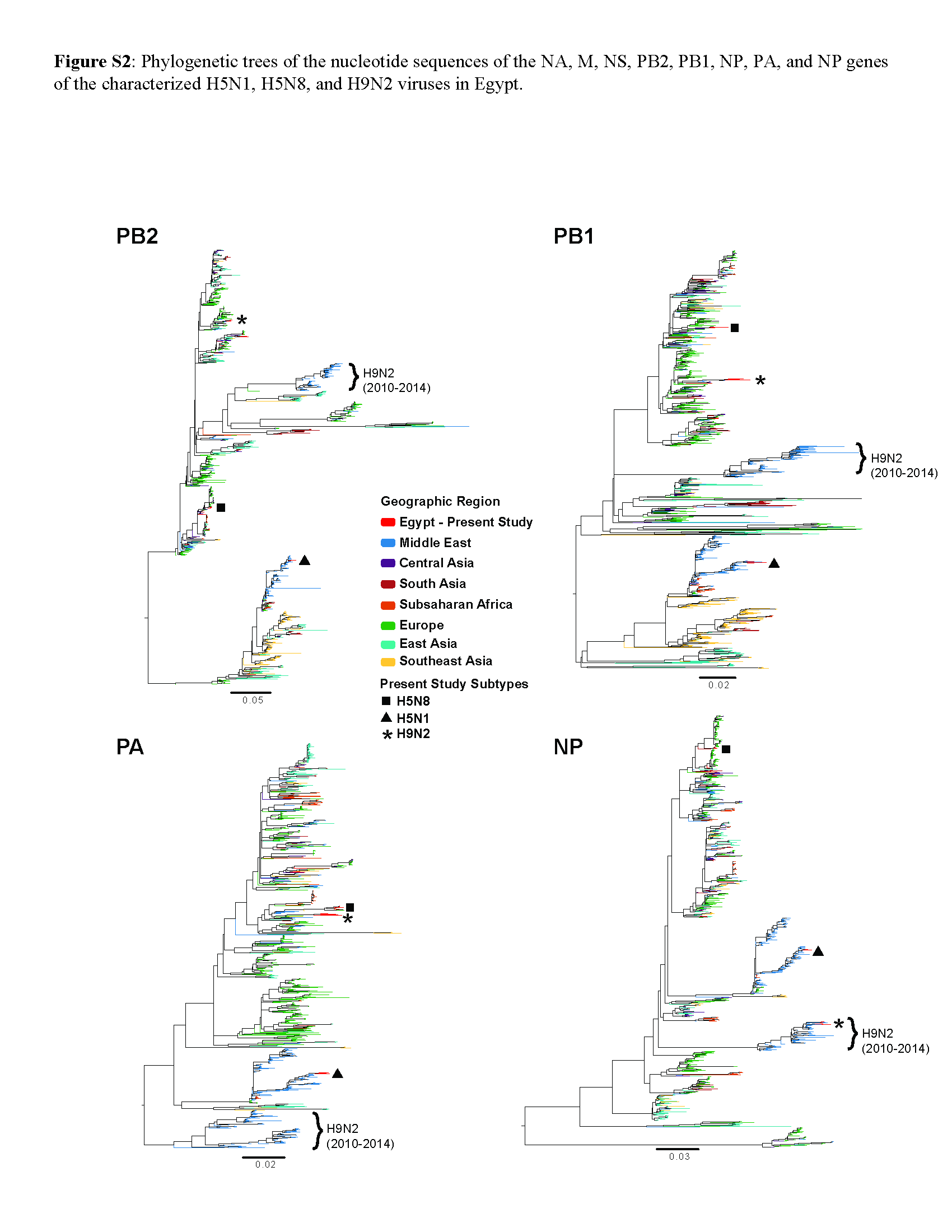

Supplement: Supplemental Material [file TEMI_A_1663712_SM3456.zip › Supplement Figure 2-page 2_final.tif]

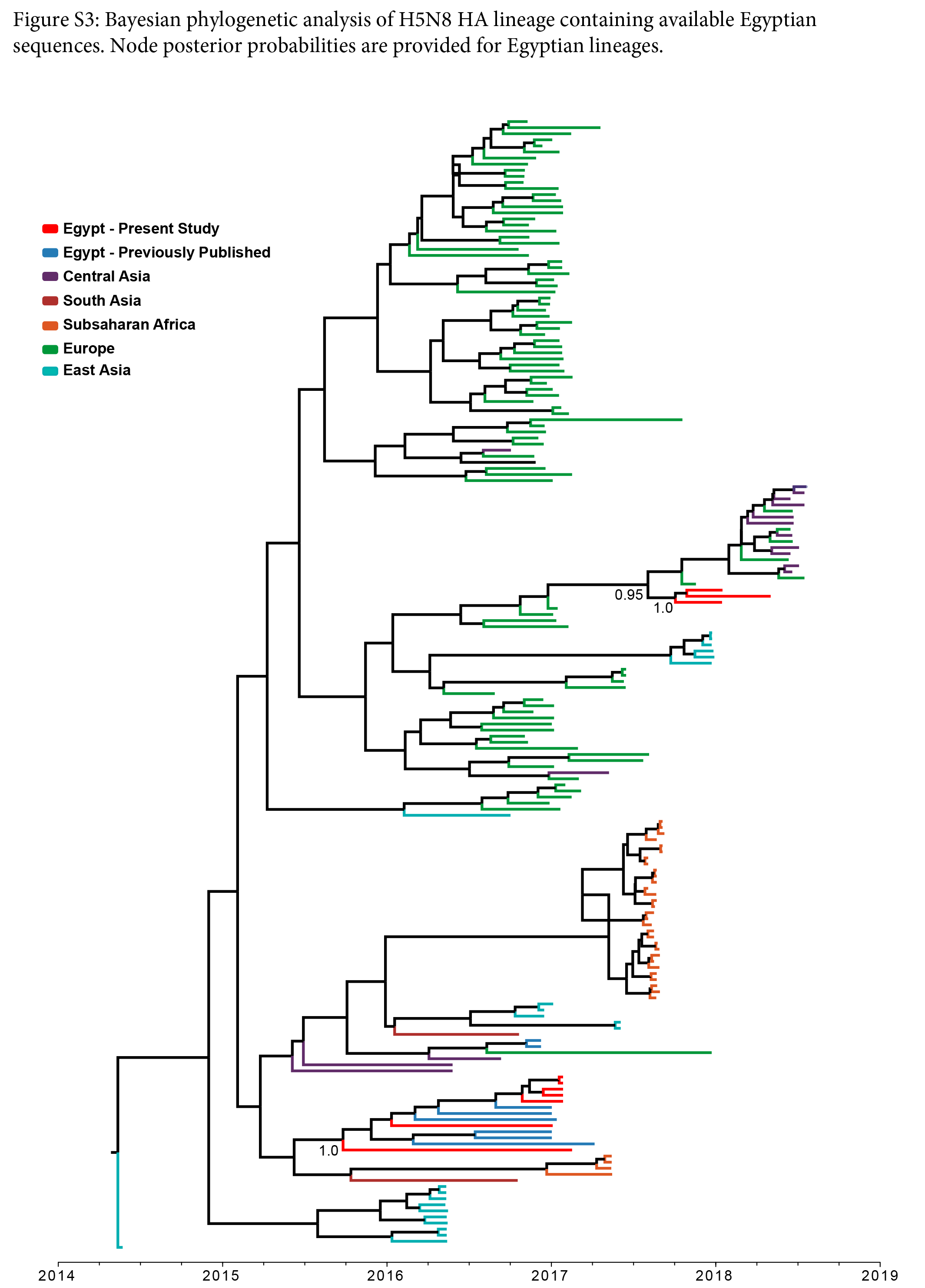

Supplement: Supplemental Material [file TEMI_A_1663712_SM3456.zip › Supplement Figure 3_final.tif]

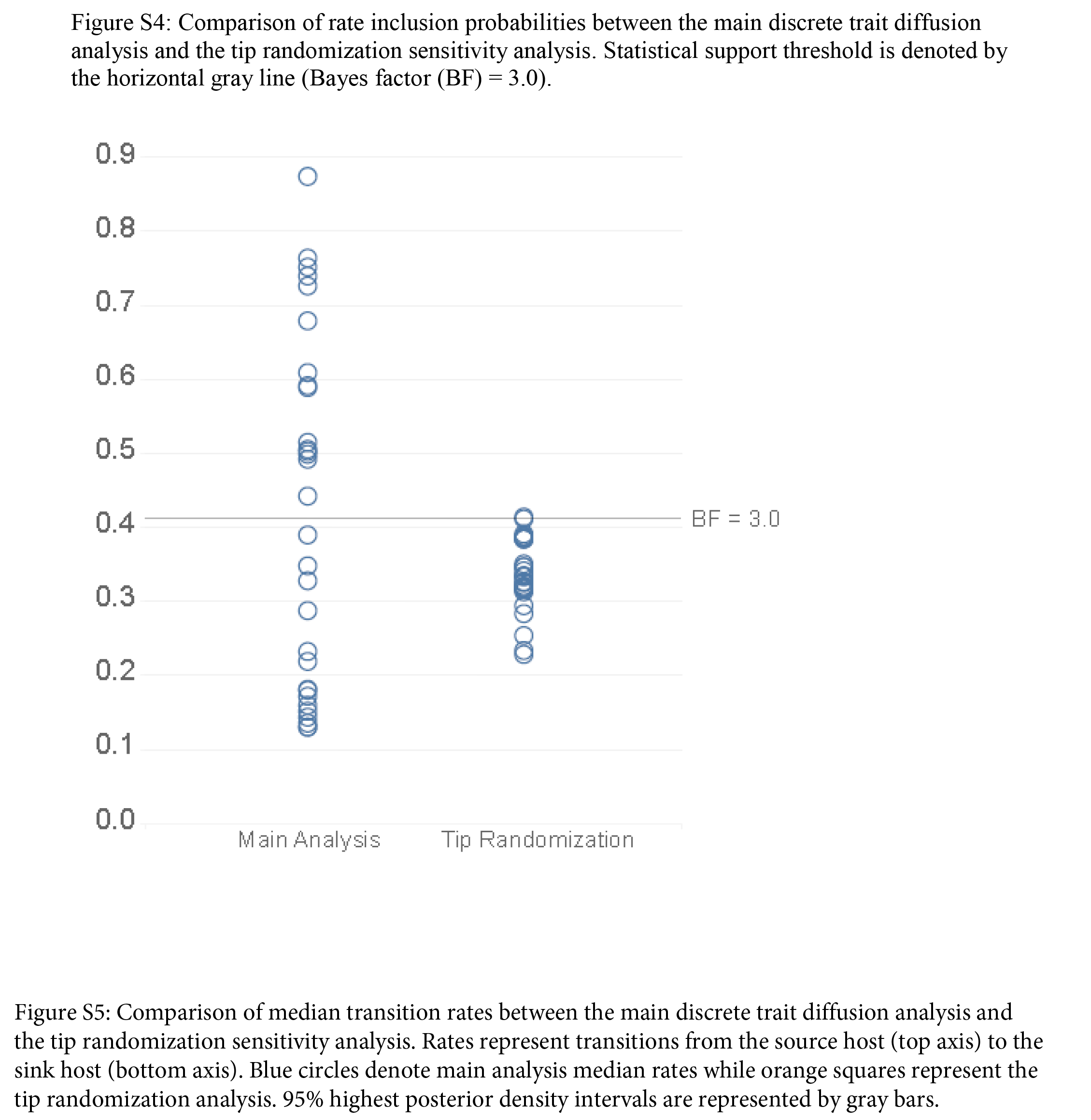

Supplement: Supplemental Material [file TEMI_A_1663712_SM3456.zip › Supplement Figure 4_final.tif]

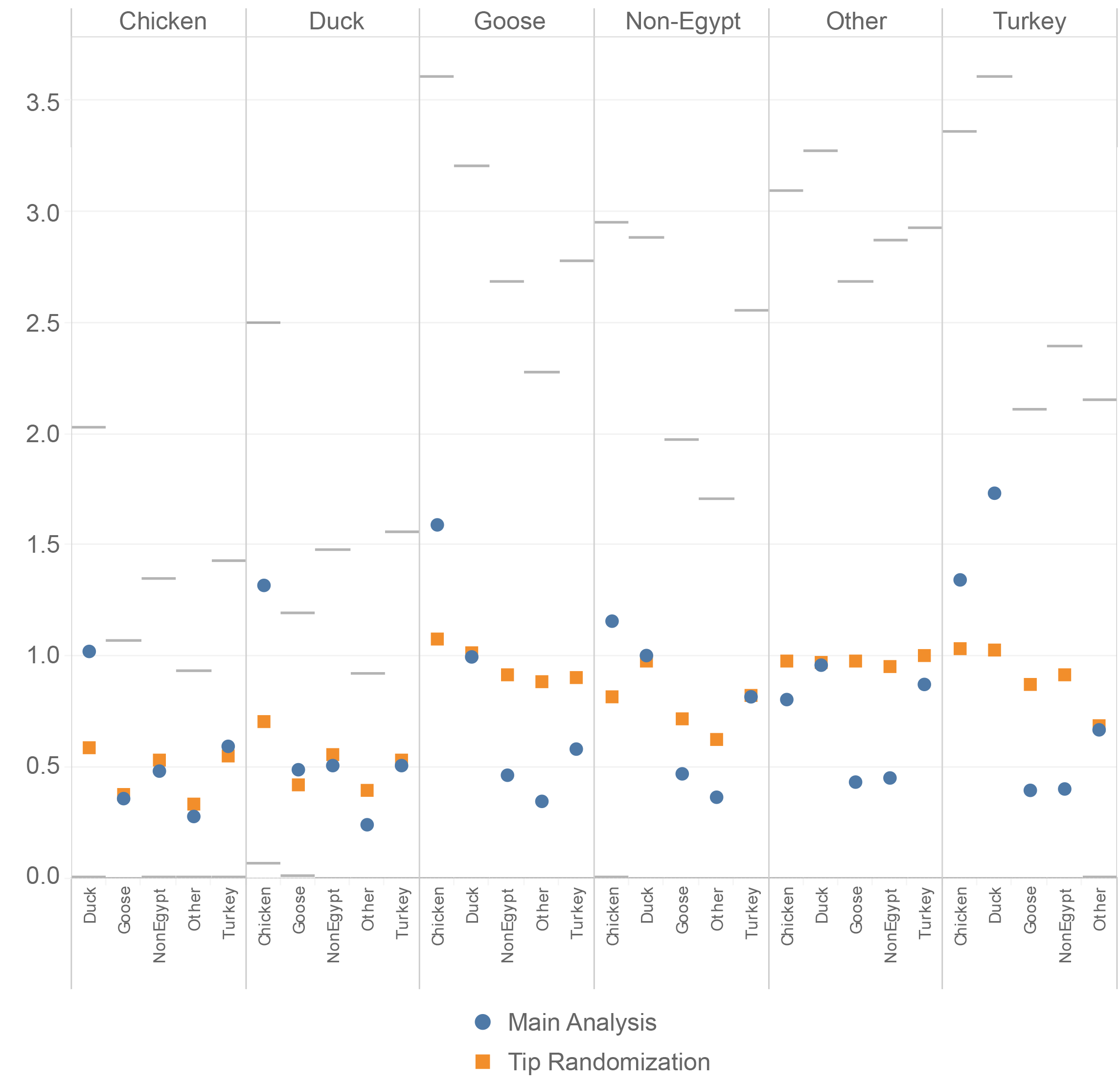

Supplement: Supplemental Material [file TEMI_A_1663712_SM3456.zip › Supplement Figure 5_final.tif]
